# Plasma metabolomic biomarkers accurately classify acute mild traumatic brain injury from controls

**DOI:** 10.1371/journal.pone.0195318

**Published:** 2018-04-20

**Authors:** Massimo S. Fiandaca, Mark Mapstone, Amin Mahmoodi, Thomas Gross, Fabio Macciardi, Amrita K. Cheema, Kian Merchant-Borna, Jeffrey Bazarian, Howard J. Federoff

**Affiliations:** 1 Translational Laboratory and Biorepository, Department of Neurology, University of California Irvine, Irvine, CA United States of America; 2 Department of Neurological Surgery, University of California Irvine, Irvine, CA United States of America; 3 Department of Anatomy & Neurobiology, University of California Irvine, Irvine, CA United States of America; 4 Department of Psychiatry and Human Behavior, University of California Irvine, Irvine, CA United States of America; 5 Department of Oncology, Lombardi Comprehensive Cancer Center, Georgetown University Medical Center, Washington, DC, United States of America; 6 Department of Biochemistry and Molecular & Cellular Biology, Georgetown University Medical Center, Washington, DC, United States of America; 7 Department of Emergency Medicine, University of Rochester School of Medicine and Dentistry, Rochester, NY, United States of America; Boston University & VA Boston Healthcare System, UNITED STATES

## Abstract

Past and recent attempts at devising objective biomarkers for traumatic brain injury (TBI) in both blood and cerebrospinal fluid have focused on abundance measures of time-dependent proteins. Similar independent determinants would be most welcome in diagnosing the most common form of TBI, mild TBI (mTBI), which remains difficult to define and confirm based solely on clinical criteria. There are currently no consensus diagnostic measures that objectively define individuals as having sustained an acute mTBI. Plasma metabolomic analyses have recently evolved to offer an alternative to proteomic analyses, offering an orthogonal diagnostic measure to what is currently available. The purpose of this study was to determine whether a developed set of metabolomic biomarkers is able to objectively classify college athletes sustaining mTBI from non-injured teammates, within 6 hours of trauma and whether such a biomarker panel could be effectively applied to an independent cohort of TBI and control subjects.

A 6-metabolite panel was developed from biomarkers that had their identities confirmed using tandem mass spectrometry (MS/MS) in our Athlete cohort. These biomarkers were defined at ≤6 hours following mTBI and objectively classified mTBI athletes from teammate controls, and provided similar classification of these groups at the 2, 3, and 7 days post-mTBI. The same 6-metabolite panel, when applied to a separate, independent cohort provided statistically similar results despite major differences between the two cohorts. Our confirmed plasma biomarker panel objectively classifies acute mTBI cases from controls within 6 hours of injury in our two independent cohorts. While encouraged by our initial results, we expect future studies to expand on these initial observations.

## Introduction

Commonly known as concussion, mild traumatic brain injury (mTBI) is a frequently encountered neurological diagnosis in pediatric, neurologic, emergency room, military, and sports medicine practices. According to the World Health Organization, 100–300 individuals per 100,000 population seek medical attention for mTBI each year. This likely represents less than half of the actual number sustaining a mTBI however, with real estimates exceeding 600/100,000 population, and thereby surpassing 40 million estimated mTBI cases worldwide each year [1]. For civilians in the United States (US) and around the world, falls represent the most common etiology associated with mTBI [1, 2]. In the US civilian sector, sport-related mTBI produces up to 3.8 million documented injuries per year [3], providing significant concerns in amateur (e.g., National Collegiate Athletic Association, NCAA) and professional (e.g., National Football League) athletics. During the last two decades, changes in warfare practices have elevated blast (i.e., explosive) injuries to the primary causative etiology for mTBI in the US active duty military [2], and from the years 2000–2016 approximately 82% of all military TBI fell into this category [4]. At least 17% of those deployed during Operation Iraqi Freedom and Operation Enduring Freedom reported at least one mTBI, and of those reporting mTBI, nearly 60% suffered more than one [5].

For both the civilian and military sectors, a diagnostic bottleneck currently exists, necessitating the development of an accurate, objective measure of mTBI that allows rapid and accurate screening of those potentially injured. Such a diagnostic measure would reduce the underreporting of mTBI and allow more appropriate care to be delivered to concussed individuals. In addition, objective biosignatures could provide a basis for temporal assessments that could guide clinical decision-making [3], such as when to allow return to play (or return to combat) [6, 7].

Metabolomic analyses in TBI and brain injury are not new. Investigations from both animal models [8–11] and the clinic [10, 12, 13] have provided important insights into alterations of specific metabolites in brain and peripheral blood, especially lipid species. A recent gas chromatography-mass spectrometry (GC-MS) investigation on blood serum from emergency room subjects with various severities of TBI or orthopedic injuries [13] identified metabolite species whose increased abundance correlated with the severity of brain trauma and subsequent poor outcome. Although the latter investigation also included significant numbers of mTBI subjects that shared similar metabolite alterations to the more severe cases, the differences between mTBI and controls were much smaller and were not the focus of the analysis. In addition, despite replicating the discovered metabolite findings in an independent cohort of TBI subjects, several ions of interest remained unidentified or could only be annotated by their chemical class [13]. The aim of our investigation therefore was to specifically explore whether metabolomic analyses of blood plasma could provide accurate, early classification of mTBI individuals from non-concussed controls (NC). Herein we present a metabolomic biomarker panel derived from a collegiate athlete (Athlete) cohort, discovered using liquid chromatography-MS (LC-MS) technology, which was ultimately annotated and confirmed via tandem MS (MS/MS). The panel accurately classifies the concussed (mTBI) Athlete group at ≤6 hours (≤6h) post-injury from their NC Athlete teammates, and is suggestive of providing effective classification during the first 7 days following injury. The same panel of metabolites was tested in an independent, more clinically diverse external validation (External) cohort, correctly classifying TBI from NC subjects, with similar receiver operating characteristic area under the curve (ROC AUC) analysis results as the internal validation for the Athlete cohort. To our knowledge, this study provides one of the first human plasma metabolomic biomarker panels, confirmed via MS/MS, which objectively classify mTBI from NC subjects under discovery, internal validation, and external replication conditions. Our biomarker panel supports previous human blood-based metabolomic results [13] in highlighting specific alterations of lipid species following TBI. Metabolomic analyses, therefore, appear poised to supplement other “omic” analyses in helping resolve the complex pathobiology resulting from TBI. If confirmed by others, through larger replicative studies, our plasma biomarkers may provide a basis for considering targeted metabolomic assays for mTBI screening and post-injury monitoring in future civilian and military clinical investigations.

## Materials and methods

### Study and protocol approvals

For the Athlete cohort participants, the Research Subjects Review Board at the University of Rochester and Rochester Institute of Technology (Rochester) provided approval for human subject participation, and all participants provided written informed consent prior to entering the study. The Medstar Health Research Institute Institutional Review Board (IRB) approved subject participation in the Washington Hospital Center (Washington) study, for individuals providing signed informed consent. The University of Maryland Shock Trauma Center (Maryland), IRB provided approval for subject participation to consenting individuals. The Headquarters, US Army Medical Research Materials Command (Army) IRB approved participation for individuals that signed informed consent documents. Finally, all the just described external protocols and informed consent documents, as well as the comprehensive combined study protocol were reviewed and approved by IRBs at Georgetown University (Georgetown) and the University of California, Irvine (Irvine), as well as by the Department of Defense Human Research Protection Office.

### Study population

**Fig 1:**
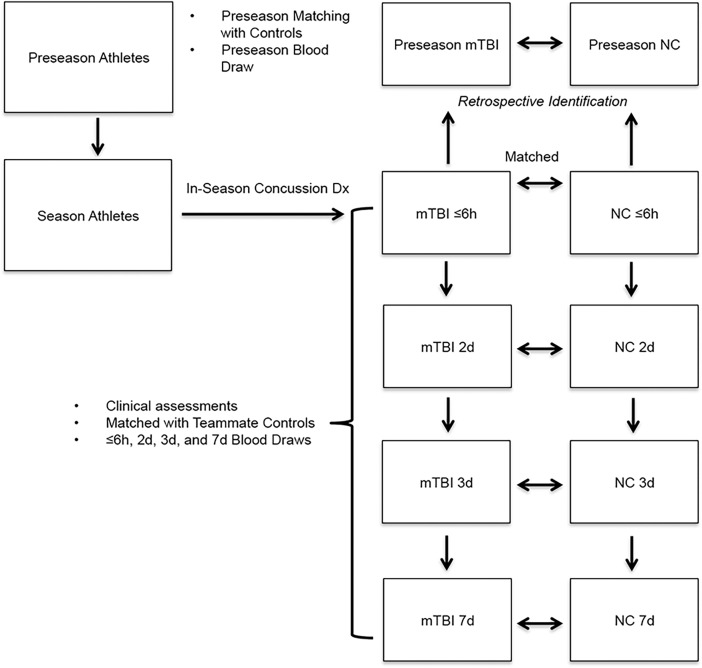
College Athlete cohort–metabolomic biomarker study design. In the college athlete cohort (Athletes), the mTBI (mild traumatic brain injury) and NC (non-concussed control) groups were definitively identified as a result of longitudinal clinical assessment of study participants throughout their sports seasons. Identification of mTBI during the Season allowed retrospective designation of group participants in the Preseason. Analytic timepoints following mTBI occurrence are indicated as **≤6h** = ≤6 hours; **2d** = 2 days; **3d** = 3 days; and **7d** = 7 days.

Our Athlete cohort (Athletes) represent a subset of 632 student participants in Division I and III NCAA contact sports, entered between 2009 and 2014 under a single Rochester sports-related mTBI protocol. Previously detailed [3], this protocol is briefly presented herein (**Fig 1**). Participants were age-, gender-, and sports-matched with teammates who would function as potential control subjects. A prior history of concussion was assessed in participating Athletes (**S1 Table**), with all meeting criteria for normal cognitive function at their Preseason baseline assessment. All Athletes underwent baseline blood sampling and cognitive testing prior to their sports season (Preseason Athletes). All Athletes were followed prospectively during their sport season (Season Athletes) and monitored for mTBI. For each mTBI case, a concussion event was initially suspected by a certified athletic trainer who witnessed the injury and performed an assessment of the subject with the aid of the Sport Concussion Assessment Tool 2 [14], and entered into the study protocol. At a later point, the final mTBI diagnosis was confirmed by a team physician using a multifaceted concussion protocol based on the most recent criteria outlined in the consensus statement on concussion in sport [15]. Only subjects confirmed by the team physician to have sustained a mTBI were included as Season Athlete mTBI cases for this study.

Season Athletes diagnosed with a mTBI underwent phlebotomy ≤6h post-injury, and then serially at 2 days (2d), 3 days (3d), and 7 days (7d) post-injury, along with their matched NC teammates who served as controls. Upon study completion, those Athletes with an mTBI diagnosis made during the Season were classified retrospectively as Preseason Athlete mTBI group, while the Season NC subjects determined the Preseason NC group.

Our External cohort subjects provided TBI and NC specimens via three distinct clinical groups participating in three unrelated and separate study protocols, allowing us to independently test any putative biomarker panel derived from the Athlete cohort. The acute TBI subjects in the External cohort took part in one of two specific neuroimaging/TBI research protocols evaluating emergency room (ER) subjects. The Washington External TBI study protocol originated from the Medstar Washington Hospital Center in Washington, DC, while the Maryland External TBI protocol took place at the University of Maryland Shock Trauma Center in Baltimore, MD. The Washington study design included blood specimens collected within 48 hours of TBI. The Washington study featured subjects with mTBI, as well as moderate or severe TBI. Blood specimens from the Maryland study were collected within 2 weeks of injury from mTBI subjects as well as more severe TBI. All NC subjects included in the External cohort were participants in an Department of Defense-sponsored investigation, from Fort Carson, CO (ClinicalTrials.gov Identifier: NCT01925963), featuring military personnel selected as controls for specific military TBI investigations, based on questionnaire responses denying a history of head injury and/or previous abnormal neuroimaging studies.

### Study procedures

We have published detailed methods regarding collection methods and metabolomic/lipidomic biomarker analyses related to preclinical Alzheimer’s dementia [16, 17] and exceptional cognitive aging [18], using both untargeted and targeted metabolomic methods [19, 20]. The current plasma analyses related to TBI utilized only untargeted metabolomic assessments, except for the tandem mass spectrometry (MS/MS) [16, 21] used to confirm the final metabolite panel.

#### Blood collection

For the Athletes (**Fig 1**), venous blood was obtained during a non-fasting state in sterile tubes containing the anticoagulant ethylenediamine-tetraacetic acid (EDTA). After thorough mixing, the tubes were placed on ice until centrifuged at 4°C (3,000 rpm for 10 minutes), within 60 minutes from venipuncture. Isolated plasma was aliquoted and stored at -80°C. Selected frozen plasma aliquots were shipped on dry ice to Georgetown for further -80°C storage until all Athlete specimens underwent metabolomic processing and analysis in a single batch. The External cohort collection protocols differed slightly from the Athlete cohort as a result of their individual study designs. Venous blood specimens from the Washington and Maryland studies were collected within EDTA tubes from non-fasting ER participants. After thorough mixing, specimen tubes were immediately packaged in wet ice for same day transport to Georgetown. For the Army study, fasting venous samples were collected in EDTA tubes from NC subjects. Specimen tubes were thoroughly mixed and placed immediately in wet ice until individually packaged with ice packs for overnight transport and delivery to Georgetown. Unfrozen specimens shipped to Georgetown on ice for analysis that arrived >24 hours from venipuncture, or without the ice or icepacks remaining cold, were rejected and not used. All study specimens collected at Georgetown as part of this study were cataloged and either stored immediately at -80°C (if frozen plasma), or processed per our published protocol [16] (if EDTA blood), with blood components isolated and stored at -80°C until further analysis.

#### Metabolomic assays

The current plasma analyses were performed in the Metabolomics Shared Resource at Georgetown, under the supervision of our co-author (AKC). Due to the disparate timing of receipt of the Athlete and External cohort specimens, metabolomic analyses were performed in two different batches, on different days, but using the same LC-MS equipment. In brief, after sequential extraction [22], untargeted metabolomic profiling of all the plasma specimens was carried out per our published protocol [16]. Metabolomic relative abundance data output was provided in two electrospray ionization (ESI) modes (negative, NEG; or positive, POS) for each analyzed sample with the analytic instrument set up to scan the 50–1200 *m/z* mass range for each ESI mode, for each plasma specimen in the data set. Each ESI mode typically provides up to 3500 unique *m/z* values. The MS raw data files are initially pre-processed using the XCMS software [23, 24] (Scripps Institute, USA). The Excel output file produced is populated with up to 3500 mode-specific *m/z* values (up to 7000 total) corresponding to putative metabolites and their relative abundances within the sample. Whereas targeted metabolomic approaches allow simultaneous detection and specific quantification of plasma metabolites in a high-throughput manner [19, 20], with current limitations to between 200 and 400 species [25], untargeted metabolomic approaches are, as described by others [26], semi-quantitative and require additional analyses for absolute analyte identification and quantification.

Since there are currently no accepted TBI-specific metabolomic biomarkers, we elected to analyze our Athlete and External cohort samples using the untargeted LC-MS approach (providing up to 7000 potential features for consideration). It is not uncommon for specimens run in different analytic batches to provide slightly different sets of *m/z* features and relative abundance values. Once putative metabolomic biomarkers are preliminarily annotated, they are either validated or rejected using available or synthesized standards via tandem mass spectrometry (MS/MS) [21] run on randomly selected case and control specimens from the original biomarker discovery cohort. Metabolites confirmed via MS/MS spectral matching are considered fully validated to a high degree of confidence.

### Outcome measures

The ability of our confirmed Athlete metabolomic biomarkers to classify mTBI cases from NC was determined at four post-injury timepoints within the Athlete cohort, at ≤6h, 2d, 3d and 7d following mTBI. The ability of the metabolite panel developed in the Athlete cohort to be generally relevant to mTBI (and TBI) diagnosis was tested in a separate, more diverse External cohort of subjects featuring a more variable severity of TBI and post-injury assessment timepoints.

### Statistical analyses

General statistical analyses were conducted with IBM SPSS (v23 for Mac, IBM, Armonk, NY, USA), and STATA/SE (v.11.2, StataCorp LP, College Station, TX, USA). Control and TBI group comparisons of age within and between cohorts were performed using independent sample t-tests. In addition to the previously mentioned software programs, we also took advantage of the Social Science Statistics website (http://www.socscistatistics.com) calculators to assist us with Chi-square (χ^2^) analyses for two groups. Cohort and group comparisons of gender, TBI severity, and time from injury to blood draw were performed using χ^2^ testing. Significance was for all statistical analyses considered at a level of p <0.05.

### Preliminary metabolite annotation

Preliminary annotation (naming) of “relevant” m/z values from the total number of features provided by the LC-MS instrument, were defined from metabolites listed within from the **Human Metabolome Database** (http://www.hmdb.ca), the **Metlin Database** (http://metlin.scripps.edu), and the **Lipid Maps Database** (http://www.lipidmaps.org), excluding common drugs and non-human metabolites. We specifically included metabolite species featured within the BIOCRATES Absolute*IDQ*® p180 Kit (Biocrates Life Sciences AG, Innsbruck, Austria), with which we have prior experience [16–18]. In this current untargeted analysis, however, we attempted to match and preliminarily annotate *m/z* features with known metabolites. To improve our preliminary annotation throughput for *m/z* values in the normalized XCMS output files received from Georgetown, we developed a proprietary web-based application, *MSF Metabolomics* (https://www.msfmetabolomics.com), that takes a formatted LC-MS metabolomic dataset Excel file, performs stepwise best matching of database-derived monoisotopic mass values, and for each ESI mode in the dataset provides a preliminary annotation for each respective *m/z* value. Best-matching using *MSF Metabolomics* is based on a user-defined matching threshold value (e.g., 0.05 or 0.01) for an accepted variability from the monoisotopic mass for the output *m/z*. The *MSF Metabolomics* output then generates a new Excel spreadsheet data file, formatted like the original and containing all the same relevant data for a reduced number of *m/z* features, but providing additional columns for each *m/z* that include best-matched annotated name, HMDB ID, Pubchem ID, and monoisotopic mass. Preliminary analytes identified are designated with the matched annotation name_ESI mode (e.g., Carnosine_N). Additional *m/z* values falling within the matching threshold of an already annotated feature are flagged and listed on a separate tab of the same new spreadsheet. The *m/z* values that are not annotatable via this best-matching approach are excluded from the new files, thereby providing an initial data reduction to relevant features based on annotation. In the current analytic process, this step significantly reduces the total number of preliminary annotated features (and *m/z* values) for analysis from a theoretical maximum of ~3500 for each mode to approximately 600 “relevant” annotated species.

### Metabolomic biomarker development

Biomarker discovery and validation/replication analyses were carried out utilizing the logistic regression (LR) and ROC AUC functions on the MetaboAnalyst 3.0 platform (http://www.metaboanalyst.ca/faces/ModuleView.xhtml) [27] and other defined analytic methods [28–30]. Input untargeted metabolomic data files, from two comparison groups, are uploaded to MetaboAnalyst 3.0 and undergo normalization using selected generalized logarithmic transformation and auto-scaling functions. Normalized data is then assessed within the Biomarker Analysis module, where the *Explorer* function provides an automated identification of significant preliminary annotated metabolites and assesses their classification performance in distinguishing the two data sets using a variety of multivariate models [31]. Specific algorithms, such as linear support vector machine (Linear SVM) [32], partial least squares-discriminant analysis (PLS-DA) [33], and random forests [34], are employed with the goal of maximizing ROC AUC using the fewest number of preliminary metabolite species. The selected algorithms within the MetaboAnalyst 3.0 platform provide a list (5–100) of significant features (variables) in predictive models. From those predictive models the performance (i.e., ROC AUC; 95% confidence interval, CI) for classifying the input phenotypic groups (e.g., cases versus controls) are provided. Biomarker panel features selected via these three unbiased statistical/machine-learning methods are then noted and tested separately for their ability to correctly classify the same two phenotypic groups using the *Tester* function of MetaboAnalyst 3.0. Within the *Tester*, all individual analytes are provided in the analytic dataset for inclusion or exclusion from the model, thereby providing the ability to define and refine biomarker panels originally derived from the *Explorer* function, that maximize classification performance. The *Tester* function rank-lists all input metabolites according to individual ROC AUC classification values, individual t-test, and fold change comparisons between the two groups being analyzed. The *Tester* function also automatically provides relevant LASSO [35] frequencies (0%-100%) for each metabolite, allowing a separate definition of an optimal LASSO-based analyte panel. Finally, model performance using selected analytes is provided using the Linear SVM, PLS-DA, random forests, and/or LR [36] algorithms. We planned comparisons of specific biomarker panel classifications between the entire Season Athlete ≤6h mTBI subjects and the Season Athlete NC subjects datasets (without splitting each into discovery and validation sets). Comparison classifications would be defined via ROC AUC values (including 95% CI, sensitivity, and specificity) derived from a LR analysis for training/discovery, and using LR with 10-fold cross validation for internal validation [37]. The optimal preliminary annotated analyte panels developed within the Athlete cohort would then undergo hypothesis-testing within the External cohort, assessing the potential for external replication of the analyte panel(s). In addition, the ≤6h Athlete cohort metabolite findings will be tested for relevant classification accuracy at later timepoints during the first week following Season Athlete mTBI, to assess classification applicability beyond the ≤6h post-mTBI timepoint. Hypothesis testing of Athlete-derived biomarker panels in the External cohort would utilize LR analyses setting the null hypothesis (H_0_) as no significant difference between External cohort TBI versus NC discovery ROC AUC results and those from the Season Athlete ≤6h mTBI internal validation (LR+ 10-fold cross validation ROC AUC) results. Additional H_0_ testing would carried out between the Season Athlete mTBI versus NC discovery LR ROC AUC results at each first week timepoint (2d, 3d, and 7d) and the internal validation results for the Season Athlete ≤6h mTBI versus Season Athlete NC groups. The Hanley-McNeil test and resulting z-statistic [28] evaluated the statistical differences between the two ROC AUC results to test H_0_, with comparisons derived using the Clinical Research Calculator for assessing the Significance of the Difference between the Areas under Two Independent ROC Curves (see Vassarstats.net).

To assess potential confounds associated with metabolomic datasets derived from different batches, a Batch Effect Adjustment module within MetaboAnalyst 3.0 allows correction of dissimilar data from otherwise similar data groups (i.e., containing both controls and cases). In our study, the two datasets tested for batch effects included the Athlete cohort, for discovery/internal validation, and External cohort, for replication [29]. Batch-corrected data is produced for each of the two datasets and allows repeat comparisons of ROC AUC results that would indicate batch-attributable differences.

## Results

### Subject characteristics

Demographic details and comparisons regarding the Athlete and External cohort participants are provided in **Table 1**. The Athlete cohort consisted of 62 subjects, including 38 that sustained a mTBI and 24 that were matched as NC. During the Preseason, 38 Athletes were designated retrospectively as being in the mTBI group while 24 were categorized as NC, providing specimens for comparative analysis. The Season Athletes, at the ≤6h timepoint, featured 27 mTBI and 24 matched NC subjects providing analytic specimens. For Season Athlete mTBI at 2d, 3d, and 7d following injury, a total of 34, 32, and 37 subjects provided specimens, respectively, while only 4 Season Athlete NC subjects provided specimens at each of the 2d, 3d, and 7d timepoints. The Athlete cohort represented those participating in basketball (*n* = 6), football (*n* = 22), ice hockey (*n* = 4), lacrosse (*n* = 4), and soccer (*n* = 26), with only ice hockey not providing matched NC subjects to those sustaining mTBI. From the Athlete participants a total of 228 plasma specimens were obtained and analyzed, including from a single Preseason and four Season timepoints. A total of 84 subjects provided single specimens for the External cohort, including 31 TBI and 53 NC subjects. The Athlete and External cohorts featured significant between-cohort differences in age for their case and control groups (p <0.05, 2-tailed independent t-test), with both Athlete cohort groups being younger than the respective External cohort groups by approximately 8 years (**Table 1**). There were no significant age differences between Athlete mTBI and Athlete NC subjects, or between External TBI and External NC subjects. There were also no significant sex differences between the two cohorts, with the Athlete cohort consisting of 31 females and 31 males and the External cohort consisting of 34 females and 50 males. While there were no significant sex differences within the Athlete mTBI, the Athlete NC, and External NC groups, significantly more males than females were represented in the External cohort TBI group (χ^2^ = 14.23; p <0.05). Sex comparisons between Athlete mTBI and External TBI groups, as a result, showed a significant difference (χ^2^ = 5.44; p <0.05). No significant sex differences were noted between Athlete NC and External NC subjects. Injury severity was significantly different between the Athlete and External cohorts (χ^2^ ≈ 13.18; p <0.05), where all 38 injured Athletes had sustained a mTBI (by study definition), while of the 31 External cohort TBI subjects, 20 (65%) were classified as having a mTBI, and 11 (35%) as more severe brain injuries (**Table 2**). Finally, the mean time to blood draw was significantly different between the Athlete TBI group used for biomarker discovery (Season Athlete ≤6h mTBI group) and that noted for the External cohort TBI subjects (χ^2^ ≈ 44.30; p <0.05) (**Table 2**).

**Table 1:** Collegiate Athlete cohort and external validation cohort demographics.

Cohorts (*n*)	Athlete (62)		External (84)		
Groups (*n*)	mTBI (38)	NC (24)	TBI-Washington (22)	TBI-Maryland (9)	NC-Army (53)
**Male/Female (*n*)/(*n*)**	22/16	9/15	18/4	8/1	24/29
**Age Range/Mean Age (years)**	18.0–22.9/19.2	18.0–21.6/18.7	19.0–35.0/27.3	19.0–35.0/26.8	18.0–35.0/27.7
**Mean Age (years) Male/Female**	19.3/19.1	18.6/18.8	27.8/25.0	26.3/31.0	26.1/27.7
**Group Type**	College Athlete	College Athlete	Civilian ER	Civilian ER	Military

**Athlete** = college athlete cohort. **TBI** = traumatic brain injury; **mTBI** = mild TBI; **NC** = non-concussed controls; **Washington** = Washington Hospital Center; **Maryland** = University of Maryland Shock Trauma Center; **Army** = NORMAL study; **ER** = emergency room.

**Table 2:** External validation cohort—TBI severity and time to blood draw.

	mTBI	mTBI*	Moderate TBI	Moderate TBI*	Severe TBI
**TBI-Washington (*n*)**	13	3	2	2	2
Mean time to blood draw (hrs. ± SEM)	15.7±2.8	13.4±3.2	32.9±8.5	34.4±4.6	23.9±11.0
**TBI-Maryland (*n*)**	7				2
Mean time to blood draw (hrs. ± SEM)	126.9±33.0				204.0±12.0

**TBI** = traumatic brain injury. **mTBI** = mild TBI. **Washington** = Washington Hospital Center. **SEM** = standard error of the mean. **Maryland** = University of Maryland Shock Trauma Center. mTBI* or TBI* = With MRI abnormality present.

### Discovery and internal validation of Athlete cohort biomarker panels

Examination of total ion chromatograms (TICs) showed a near perfect overlay (**S1 and S2 Files**), with minimal drift in retention times in both ESI modes for the Athlete cohort discovery and internal validation analytic sets. The coefficient of variation (CV) for the internal standards used in the analyses was <15%. The mass accuracy was within 7 parts per million (ppm) over the mass range of 50–1200 Daltons throughout the batch acquisition.

**Fig 2:**
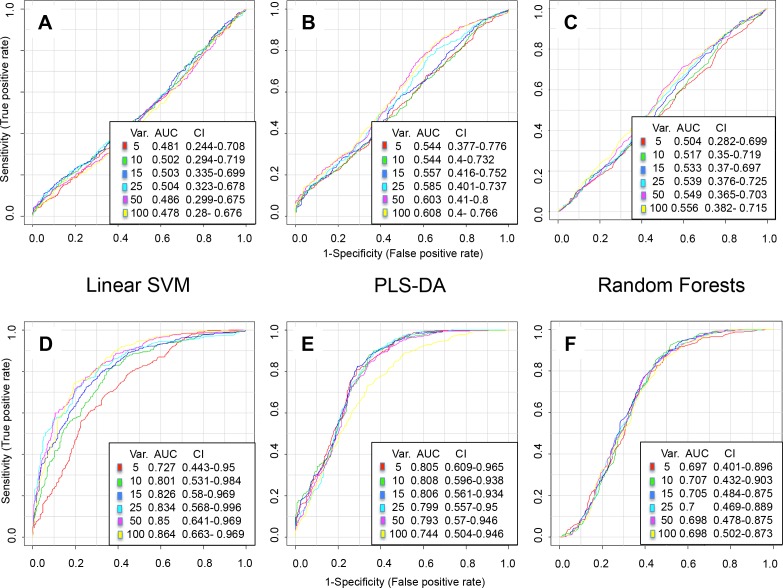
Athlete cohort preliminary multivariate ROC AUC analysis plots. Example college athlete cohort (Athletes) receiver operating characteristic (ROC) area under the curve (AUC) analysis results from the MetaboAnalyst 3.0 *Explorer* function of Biomarker Analysis module, with plots of sensitivity (y-axis) and 1-specificity (x-axis). In **A-C**, the plots indicate no significant difference between the CAC Preseason mild traumatic brain injury (mTBI) and non-concussed subjects (NCS) groups using either (i) Linear support vector machine (SVM), (ii) partial least squares discriminant analysis (PLS-DA), or (iii) random forests methods. ROC AUC values in all three analyses are ~ 0.5. The legend at lower right of each graph indicates AUC and 95% confidence interval (CI) values for derived models using 5–100 analytes (Var.). Plots **D-F** provide examples of more significant differences between comparison groups, such as between Athlete Preseason NC (non-concussed teammate controls) and the Season NC groups, using the same analytic methods as A-C, but with ROC AUC results ranging from about 0.70 to 0.86.

An untargeted metabolomics analysis of the Season Athlete ≤6h mTBI and NC subject groups provided a total of 2811 distinct XCMS *m/z* features for consideration in the biomarker analyses, with 1422 from the NEG mode and 1389 from the POS mode. Preliminary annotation of the NEG and POS mode data with *MSF Metabolomics* resulted in a reduction to 294 annotated metabolite species, with 82 and 212 from the NEG and POS modes, respectively. An initial comparison of the Preseason Athlete NC and mTBI groups, utilizing all 294 metabolite species, confirmed that there were no significant analyte differences between the groups, using each of the three different multivariate analytic approaches that provided ROC AUC results of ~0.50 **(Fig 2A–2C)**. This confirmed the initial metabolomic similarity between the Preseason Athlete groups. Using the same analytic algorithms, similar ROC AUC results (**Fig 2A–2C**) were determined when evaluating specimens from the Season Athlete ≤6h NC subjects (n = 24) and those from the combined Season Athlete NC subjects (n = 12) at the 2d, 3d, and 7d timepoints (data not shown). The lack of major metabolite differences between the Season NC timepoints supported their combination into a single Season Athlete NC group (*n* = 36) for comparison with Season Athlete mTBI subjects at each of the post injury timepoints (≤6h, 2d, 3d, and 7d).

Group differences did exist, however, between the Preseason Athlete NC and the Season Athlete NC (combined ≤6h, 2d, 3d, 7d) groups (**Fig 2D–2F**), with similar results when comparing the Preseason Athlete mTBI and the Season Athlete mTBI groups (data not shown), with the latter group tested at each of the post-injury timepoints (≤6h, 2d, 3d, 7d). We have yet to confirm an explanation for the apparent differences between Preseason and Season Athlete plasma metabolite differences noted between NC and mTBI groups. We reasoned, however, that such differences necessitated our comparison of only metabolites from Season Athlete mTBI and NC subjects and exclude those from the Preseason in our biomarker development process.

The initial number of Athlete cohort mTBI analytes (variables) selected for testing in our classification algorithms was based on our previous experiences [16–18]. We decided, *a priori*, to initially include the top ten (10) analytes provided by the discovery feature selection process. Within this investigation, we had noted that top 10 variables had provided adequate estimates of the top multivariate AUC result, provided by between 5 and 100 variables (**Fig 2A, 2C, 2E and 2F**). Refinement of the number of variables (metabolites) to include and test in a final classification model would be made as necessary. Within the Athlete cohort discovery dataset, therefore, we sought to discover an optimal metabolite panel using consistent and conservative statistical and machine learning approaches for feature selection. The feature selection methods used included LASSO and five other approaches to develop six distinct preliminary panels (**S2 Table**). Application of the six panels within the MetaboAnalyst 3.0 *Tester*, and deriving LR-based ROC AUC results for comparison indicated in the **S2 Table** (shaded cells) that the best discovery and internal validation results were provided by the Linear SVM and LASSO feature selection methods. Both models consisted of 10 metabolite panels that provided superior training/discovery ROC AUC results. With nearly identical classification results, both of these derived 10-metabolite panels were then tested on the preliminarily annotated External cohort samples, to examine their potential for classifying the TBI from NC groups. The remaining panels were excluded from further consideration.

Attempted matching of preliminary annotations from the Athlete cohort Linear SVM- and LASSO-derived 10-metabolite panels to similar metabolites in the External cohort resulted in an incomplete match, with only six of ten Linear SVM metabolites common to both cohorts, and only eight of ten from the LASSO panel (**Table 3**). Both original Linear SVM and LASSO panels featured primarily lipid species.

**Table 3:** Discrepant best preliminary analyte panels between athlete and external cohorts.

Linear SVM 10	LASSO 10
LysoPI a C20:5_N	13-HODE_N
PS aa C42:6_N	LysoPA a C22:6_P
13-HODE_N	DAG aa C33:2_P
FA C18:0_N	PG ae C33:3_P
AC16:2_P	LysoPC a C20:4_P
FA 2-OH C16:0_N	PC ae C34:4_N
LysoPA a C16:0_N	TUDCA_N
LysoPA a C15:1_N	FA C28:7 n-6_P
Asparagine_N	PE ae C36:4_N
Carnosine_ N	PE aa C38:6_N

Shaded cells indicate preliminary annotated analytes common to both Athlete and External cohorts from the original 10-metabolite panels derived using Linear SVM and LASSO feature selection. **SVM** = support vector machine. **LASSO** = least absolute shrinkage and selection operator. **AC** = acylcarnitine. **DG** = diacylglycerol. **FA** = fatty acid. **LysoPA** = lysophosphatidic acid. **LysoPC** = lysophosphatidylcholine. **LysoPI** = lysophosphatidylinositol. **PC** = phosphatidylcholine. **PE** = phosphatidylethanolamine. **PS** = phosphatidylserine. **TUDCA** = tauroursodeoxycholic acid. **HODE** = hydroxyoctadecadienoic acid. Note that 13-HODE_N is present in both original panels.

Retesting the ability of common analyte panels (Linear SVM 6 and LASSO 8) to classify groups in the original Athlete cohort discovery datasets (Season Athlete ≤6h mTBI versus Season Athlete NC) provided comparable discovery and internal validation results to those obtained with the original 10-member panels (**Table 4**).

**Table 4:** Comparison of unmatched and matched biomarker panel results between Athlete and external cohorts.

Feature Selection Method	Number of analytes in panel (*n*)	Athlete Cohort Training/Discovery ROC AUC (95%CI) (sens/spec)	Athlete Cohort Internal Validation ROC AUC (95% CI) (sens/spec)	External Cohort Replication ROC AUC (95% CI) (sens/spec)	Hanley- McNeil Test Results (z, p)
**Linear SVM**	10	**0.976** **(0.965–0.988) (**0.778/1.00**)**	**0.864** **(0.750–0.978) (**0.815/0.917**)**		
	6	**0.913** **(0.888–0.938)** (0.835/0.907)	**0.851** **(0.745–0.957)** (0.815/0.861)	**0.830** **(0.798–0.861)** (0.817/0.715)	**0.293, 0.770**
**LASSO**	10	**0.974** **(0.963–0.985)** (0.663/1.00)	**0.865** **(0.771–0.960)** (0.704/0.944)		
	8	**0.948** **(0.930–0.965)** (0.852/0.914)	**0.848** **(0.746–0.949)** (0.741/0.861)	**0.811** **(0.781–0.841)** (0.778/0.686)	**0.502, 0.616**
**MS/MS**	6	**0.847** **(0.815–0.879)** (0.770/0.784)	**0.791** **(0.677–0.905)** (0.741/0.778)	**0.738** **(0.703–0.773)** (0.695/0.644)	**0.633, 0.527**

Shaded areas highlight same row comparison group results used to test the null hypothesis (H_0_) via the Hanley-McNeal Test, that no significant difference exist between CAC and EVC ROC AUC results. **Training/Discovery** = uses logistic regression analysis. **CI** = confidence interval. **ROC AUC** = receiver operating characteristic area under the curve. **sens/spec** = sensitivity/specificity. **Internal Validation** = uses logistic regression with 10-fold cross validation analysis. **Replication** = uses logistic regression analysis, similar to Training/Discovery. **MS/MS** = preliminary metabolites from Linear SVM 6 and LASSO 8 panels definitively confirmed using tandem mass spectroscopy (MS/MS). **z** = Hanley-McNeil Test statistic. **p** = 2-tailed level of significance. Statistical significance considered if p <0.05.

#### Replication of biomarker panels in an external cohort are confirmed

The External cohort specimens provided a total of 2518 distinct *m/z* features for biomarker analysis, with 1221 in NEG mode and 1297 in POS mode. Preliminary annotation using *MSF Metabolomics* resulted in data reduction to a total of 435 (206 NEG and 229 POS) metabolites for biomarker testing. While differences in Athlete and External cohort demographics, injury severity, time to blood draw, and separate batch processing clearly raised the probability for discrepant group classification results between the cohorts, we did not find this to be the case. Within the External cohort dataset we tested whether to accept or reject the H_0_, that no significant differences existed using either of the two common biomarker panels (Linear SVM 6 or LASSO 8), when comparing the Athlete cohort internal validation results and External cohort’s replication results, as in **Table 4** (shaded cells). No statistical difference in ROC AUC results is noted when direct comparisons are made using the Hanley-McNeil Test [28], displayed in **Table 4** (far right column). By accepting the H_0_, therefore, the classification capability of both preliminary biomarker panels met criteria for external replication, despite the previously defined differences between the two cohorts.

### Biomarkers are confirmed using tandem mass spectrometry in both cohorts

The final confirmation of the molecular identities for metabolites originating from the Linear SVM 6 and LASSO 8 panels, common to both Athlete and External cohorts, was undertaken via MS/MS. Of the original 13 distinct metabolites in the two combined panels (one was common to both), six metabolite species received confirmatory annotation via MS/MS (**Table 5**). The specific MS/MS fragmentation pattern for each of the six metabolites (**S3 File**) were compared and confirmed with those of spectra from available standards within the Human Metabolome or Lipid Maps databases, as we previously reported [38]. Comparison of the final MS/MS 6 panel with the previous preliminary panels disclosed only a slight loss of classification accuracy in the Athlete cohort and maintenance of replication within the External cohort, as noted in **Table 4** (bottom row). The MS/MS 6 mTBI-derived metabolite panel, therefore, is confirmed under discovery, internal validation, and external replication conditions using two independent subject cohorts.

**Table 5:** Final MS/MS-confirmed biomarker panel analyte details.

Analyte	FA 2-OH C16:0	FA C18:0	TUDCA	PE ae C36:4	PE aa C38:6	LysoPC a C20:4
**Other Name**	2-hydroxypalmitic acid; 2-hydroxy-hexadecanoic acid	Stearic acid; Octadecanoic acid	Tauroursodeoxy-cholic Acid	PE (P-16:0/20:4); Phosphatidylethanolamine Plasmalogen	PE (16:0/22:6); Diacyl-Phosphatidyl-ethanolamine	PC (20:4/0:0); Lyso-phosphatidylcholine
**ESI Mode**	NEG	NEG	NEG	NEG	NEG	POS
**Level With TBI**	Down	Up	Down	Up	Down	Up
**m/z value**	271.2266	283.2629	498.2936	722.513	762.5081	544.3411
**Monoisotopic mass value**	272.235	284.272	499.2968	723.5203	763.5152	543.3325
**Pubchem ID**	92836	5281	9848818	52925126	9546799	24779476
**HMDB ID**	HMDB31057	HMDB00827	HMDB00874	HMDB11352 HMDB11353	HMDB08946	HMDB10395
**LM ID**	LMFA01050047	LMFA01010018	LMST05040015	LMGP02030093	LMGP02010095	LMGP01050048

Biomarkers derived from the Athlete cohort and replicated in the External cohort. **MS/MS** = tandem mass spectrometry. **FA** = fatty acid. Phospholipid designations include: typically, **ae** = ether bond (**e**) at sn1 position and ester acyl bond (**a**) at sn2 position. Ether bonds are either alkyl (ether, O-) or alkenyl (plasmalogen, P-). Lipid species nomenclature features C (number of carbons):(number of double bonds). Phospholipids feature glycerol conjugated fatty acyl, alkyl, or alkenyl species designated to sn1/sn2 positions (e.g., C16:0(sn1)/22:6(sn2)). **ESI** = electrospray ionization mode. **TBI** = traumatic brain injury. **m/z** = mass/charge. **HMDB ID** = The Human Metabolome Database identification (www.hmdb.ca). **Pubchem ID** = Open Chemistry Database identification (https://pubchem.ncbi.nlm.nih.gov/). **LM ID** = Lipid Maps Lipidomics Gateway identification (www.lipidmaps.org).

#### Validated panel possibly useful with more severe TBI and in delayed mTBI diagnosis during the first week following injury

Interestingly, we found evidence that the ability to classify the TBI from NC groups using the MS/MS 6 panel is not significantly different between the Athlete cohort mTBI cases and the External cohort TBI cases despite the latter featuring more complex/severe injuries (**S3 Table**). We extended our assessment of the Athlete-derived preliminary and MS/MS-confirmed biomarker panels to include the first week following mTBI (**S4 Table**), again testing the H_0_, regarding whether biomarker panel provided similar classification results at the 2d, 3d, and 7d timepoints following mTBI as was originally provided at the at the ≤6h timepoint. For the two preliminary and the final MS/MS confirmed biomarker panels tested, there were no significant classification differences noted during the Season Athlete group’s first week timepoints, based on Hanley-McNeil analyses. Semi-quantitative relative value (RV) plots for the six MS/MS-confirmed metabolites (**S1 Fig)** in Season Athletes over the first week following mTBI show no significant individual differences.

#### Batch correction does not significantly alter discovery/internal validation and replication results

Assessing the Athlete cohort (≤6h mTBI versus Season NC) and the External cohort (TBI versus NC) data, prior to and following Batch Correction, provide evidence via principal component analyses (PCA) (**S2 Fig)** of differences between the two cohorts that can be ameliorated via the data adjustment. When assessing semi-quantitative RV plots for the two cohorts, from before and after Batch Correction, the varying abundances between the same metabolites in the two cohorts prior to Batch Correction (**S3A and S3B Fig**) are appreciated. As a result of the Batch Correction Adjustment (**S3C and S3D Fig**) there is a noticeable improvement in the comparability of the metabolite abundance data, while not eliminating specific cohort differences (as previously described). A repeat ROC AUC analysis for discovery, internal validation, and external replication, before and after the Batch Correction Adjustment (**S5 Table**) indicates no significant differences in ROC AUC results for each of the preliminary biomarker panels and for the final MS/MS 6 panel.

## Discussion

The alterations of certain measurable blood proteins continue to receive the major focus of experimental and clinical TBI biomarker research since the 1980s [39–50], despite their kinetics of expression [51] making them difficult (moving targets) to develop as reliable diagnostics. Additional limitations to relevant blood-based proteomic assay development include a combination of inherent (genetic, etc.) and technical (collection and processing) variabilities [52, 53], as well as constraints related to assay-imposed detection limits. With some recent exceptions, these factors continue to provide significant constraints on the development of proteomic-based TBI diagnostics, especially for mTBI.

Reliable, objective, minimally invasive biomarkers for mTBI would be immediately impactful to the practice of civilian and military medicine. The objective diagnosis of mTBI would enable earlier and more specific treatment options to be considered and initiated. Likewise, novel mTBI biosignatures might permit serial monitoring of individuals during their recovery, affording healthcare providers objective evidence of recuperation, in support of return-to-play (or return-to-fight) determinations [6, 7] or may herald impending post-concussive sequelae [54] requiring additional treatment or monitoring. The latter clinical distinctions are more relevant today than ever, as we better appreciate the consequences of multiple concussive [55] and subconcussive [56] injuries in the etiopathogenesis of the neurological consequences that may follow mTBI.

The biomarker discovery methods chosen for our study took advantage of closely matched teammates within the Athlete cohort in attempting to differentiate potential metabolomic differences directly related to mTBI. The total number of subjects used in our Athlete cohort’s discovery group (Season Athlete ≤6h mTBI and NCS subjects) was projected *a priori* to provide adequate power for a classification ROC AUC of 0.70 at the .05 significance level. In actuality, the Athlete cohort internal validation ROC AUC result of nearly 0.80 exceeded those projections. At the discovery phase of biomarker identification, there is always the potential that a discovered biomarker panel is overfit to the particular discovery cohort used to generate it. In our study, such overfitting is less likely since to the same biomarker panel provides comparable classification, as displayed in **Table 4** (bottom row), in an independent External cohort which features subjects with different age, severity of injury, and time to blood draw, and with specimens run in a different batch from those for the discovery (Athlete) cohort.

Our metabolomic results support those of others [13], adding to evidence that mTBI-related alterations in specific blood metabolite abundances occur early and may persist during the first week following injury. Case/control classification during the first week following mTBI using metabolomic biomarkers may increase the accuracy and rapidity of diagnosis, may influence therapeutic choices, and based on specific metabolites [13] may offer prognostic significance. Important oxidative changes are known to occur within minutes to hours following mTBI in the brains of rodents [57] and humans [58], and have also been reflected in the periphery [59]. Systemic molecular species that may mirror brain lipid peroxidation and antioxidant levels following experimental TBI, however, have typically recovered to baseline after 48 hours [58, 59]. Orešič and colleagues [13], however, have reported elevations in two medium chain fatty acids (C8 and C10) during the first week following moderate and severe TBI. While three metabolites in our confirmed plasma biomarker panel show elevated relative abundance values (**Table 5, S1 Fig, and S3A and S3C Fig**) at the Season Athlete mTBI ≤6h timepoint compared to NC, there are slight abundance discrepancies within the External cohort (**S3B and S3D Fig**). The remaining three MS/MS-confirmed metabolites express reductions in abundances following mTBI in our Season Athletes. Although similar metabolite alterations in CSF might pose a less daunting interpretation, our confirmed biomarkers might represent either CNS-specific and/or non-CNS expressions following at least an mTBI. Additional investigations beyond this current study are required to confirm whether our biomarker panel reflects confounds associated with non-CNS trauma. The addition of non-CNS injury controls (i.e. orthopedic injuries) has been effectively utilized [13], and will be essential to a more complete interpretation of our metabolomic results in future investigations. Despite this limitation of our current study, we are encouraged by the fact that the Season Athlete NC subjects were otherwise closely matched to their Season Athlete mTBI teammates, enduring similar sport-related workouts and sustaining comparable non-CNS trauma during their sports season. The latter allows us to suggest, therefore, that metabolomic differences between the groups of teammates are more than likely related to the mTBI than other group differences. The extended classification applicability of our Athlete mTBI metabolite panel during the first week following injury needs further replication with larger numbers of subjects. Larger sample sizes at all timepoints following mTBI will help clarify specific metabolite fluctuations suggested by our proposed biomarkers (**S1 Fig**) during the first week following injury. Interestingly, the MS/MS 6 panel provided similar classification in the External cohort for TBI and NC subjects, despite the varying injury severity and assessments at more variable and prolonged post-injury timepoints.

Despite having an unconfirmed CNS origin, our six plasma biomarkers (**Table 5**) appear causally and temporally associated with mTBI. The metabolite 2-hydroxypalmitate, for example, is typically generated by fatty acid 2-hydroxylase (FA2H) [60], either in association with α-oxidation of odd-chain fatty acids [61] or in generating the 2-hydroxy fatty acids for incorporation into sphingolipids [62], including myelin. Since galactosylceramide and sulfatide comprise approximately 25% of myelin lipids [63], and more than 50% of these sphingolipids in myelin feature 2-hydroxylated fatty acids [64], reduced levels in plasma may indicate post-injury flux into the CNS [17, 65], possibly in an attempt to repair white matter injuries commonly associated with mTBI [66]. Palmitic acid (palmitate) is the most common saturated fatty acid in human plasma [67]. Stearic acid, the second most common saturated fatty acid in human plasma [67] is known to increase in rodent brain following a controlled cortical impact with blood-brain-barrier disruption [68], and could consequently be elevated in blood plasma following TBI. Sphingolipids [10] and medium-chain fatty acids [13] have been reported to increase in blood following TBI and stroke, with the fatty acid species possibly reflecting mitochondrial failure associated with TBI [12]. The taurine conjugated bile acid, tauroursodeoxycholic acid (TUDCA), has been shown to be neuroprotective in humans through the prevention of apoptosis and other pathobiologic cascades in a variety of human neurological disorders, including TBI [69]. Decreased plasma levels of TUDCA, therefore, could possibly be associated with more detrimental effects following mTBI, although the mechanism associated with the observed reduction is yet to be defined. Brain glycerophospholipids typically have unsaturated or monounsaturated 16 or 18 carbon fatty acids (or fatty alcohols) at the sn-1 position [70], as in our panel’s two phosphatidylethanolamines (PEs). The sn-2 position, especially in plasmalogens (e.g., our P-16:0 species) usually features either arachidonic acid (AA, C20:4) or docosahexaenoic acid (DHA; C22:6), providing a pool of second messenger precursors for release from membrane phospholipid pool via phospholipase A_2_ (PLA_2_), especially in cortical gray matter [71]. Altered levels of our two PE species have been previously reported in rodent plasma up to 3 months following TBI [72], possibly in association with persistent generation of brain AA- and DHA-derived second messengers. Finally, in experimental brain trauma, lysophosphatidylcholine (lysoPC) levels are known to increase above normal levels in CSF for up to 6 days following TBI [73], primarily as a result of PLA_2_ activation. In plasma, however, lysoPCs are used to transport polyunsaturated fatty acids (PUFAs) to various tissues (including brain) [74]. Elevations in plasma lysoPCs containing AA, as in our study, might reflect a compensatory response to increased demand for AA membrane precursors as a result of TBI and the enzymatic or oxidative removal of such a PUFA from the brain’s Lands’ Cycle [75, 76]. Alternatively this observation could reflect abnormalities in the concussed brain’s ability to take up such lysoPC species from blood, as has been associated in some humans the carrying apolipoprotein E ε4 allele [77]. Determining the underlying mechanisms responsible for our specific plasma biomarkers (and those of others investigators) may ultimately reflect on specific pathobiologic mechanisms associated with mTBI.

The presence of plasma metabolite signals that accurately classify mTBI from NC subjects may help spur the development of next generation metabolomic technologies that are no longer dependent on MS (or NMR). Parallel novel diagnostic tools are currently being advanced for a number of TBI-associated proteomic biomarkers [78]. Such efforts are likely to portend point-of-service (POS) products capable of rapid, objective mTBI diagnosis in the ER, the sports field, and the battlefield. We anticipate advancing our mTBI metabolomic investigations to assess the CNS-specificity of our confirmed biomarkers in other mTBI cohorts while exploring novel diagnostic technologies.

## Supporting information

S1 Fig MS/MS-confirmed metabolite panel fluctuations over first week following mild traumatic brain injury in the athlete cohort. Individual mean metabolite relative values (RVs) ± SEM for Athlete NC and mTBI timepoints (≤6h, 2d, 3d, 7d) are plotted for each of the MS/MS confirmed metabolites. No statistically significant changes from NC values are noted for each metabolite, despite some fluctuations. NC = non-concussed controls. SEM = standard error of the mean. MS/MS = tandem mass spectrometry.
(TIF)

S2 Fig Principal component analysis plots of athlete and external cohort datasets before and after batch correction adjustment. Note the dense clustering of Athlete cohort data in PC1 prior to Batch Correction (left) compared to after (right). Both Athlete and External cohorts appear more evenly distributed based on PC2, before and after adjustment. Although the Athlete and External cohort data overlap is improved with the Adjustment, the datasets continue to show differences.
(TIF)

S3 Fig Pre- and post-batch correction relative abundances for the MS/MS-confirmed biomarkers. Mean relative values (RVs) ± SEM for NC and mTBI groups in the Athlete cohort at the ≤6h timepoint (A & C) and for the External cohort TBI and NC groups (B & D) are presented for each of the MS/MS-confirmed 6 plasma biomarkers. Note the relative improvement in quantitative comparability of metabolite RVs in both cohorts following Batch Correction (A vs. B; C vs. D). The individual analyte relative value (RV) differences did not reach statistical significance between the NC and mTBI (or TBI) groups, before or following batch correction. NC = non-concussed controls. TBI = traumatic brain injury. mTBI = mild TBI. SEM = standard error of the mean. MS/MS = tandem mass spectrometry.
(TIF)

S1 File Quality Control (QC) total ion chromatogram–negative mode. Athlete cohort discovery/internal validation specimen set. Note the complete QC pool overlay, and apparent consistency across all QCs.
(PDF)

S2 File Quality Control (QC) total ion chromatogram–positive mode. Athlete cohort discovery/internal validation specimen set. Note the complete QC pool overlay, and apparent consistency across all QCs.
(PDF)

S3 File The specific fragmentation spectra for each of the six MS/MS-confirmed metabolites. These six fragmentation spectra obtained from discovery specimens were matched with those known standards within the Human Metabolome or Lipid Maps Databases, using standard methods [38]. The six included spectra, therefore, confirmed our 6-metabolite panel that was discovered and internally validated within the Athlete cohort and replicated in the External cohort.
(PDF)

S1 Table Athlete cohort—prior history of traumatic brain injury. mTBI = mild traumatic brain injury. NC = non-concussed teammate control.
(DOCX)

S2 Table Athlete cohort analysis using six feature selection-derived models. Shaded areas indicate models with best results when comparing ROC AUC values using various feature selection methods in the Athlete cohort. **LR** = logistic regression. **CI** = confidence interval. **ROC** = receiver operating characteristic. **AUC** = area under the curve. **sens/spec** = sensitivity/specificity. **SVM** = support vector machine. **PLS-DA** = partial least squares-discriminant analysis. **LASSO** = least absolute shrinkage and selection operator. **Targeted 1** = selected based on highest-ranking metabolites AUC values in the *Tester* for analytes included in Biocrates *AbsoluteIDQ®* p180 Kit. **Targeted 2** = selected based on the highest-ranking lipid AUC values in the *Tester* for analytes included in Biocrates *AbsoluteIDQ®* p180 Kit.
(DOCX)

S3 Table MS/MS 6 panel classification accuracy for the TBI severity groups. Gray shaded area depicts comparison Season Athlete ≤6h internal validation values for testing the null hypothesis on External cohort Replication ROC AUC results from each of the mTBI and >mTBI groups. **CI** = confidence interval. **MS/MS 6** = Final six metabolite panel confirmed via tandem mass spectrometry (MS/MS). **ROC** = receiver operating characteristic. **AUC** = area under the curve. **sens/spec** = sensitivity/specificity. **Training/Discovery** = uses logistic regression analysis. **Internal Validation** = uses logistic regression with 10-fold cross validation analysis. **Replication** = uses logistic regression analysis. **NC** = non-concussed controls. **mTBI** = mild traumatic brain injury. **>mTBI** = TBI noted to be worse than mTBI, including mTBI with abnormal MRI, moderate TBI, or severe TBI. *No statistically significant difference when compared to shaded value in same row, via Hanley-McNeil test. Statistical significance considered if p <0.05.
(DOCX)

S4 Table Biomarker panel classification in the athlete cohort at ≤6h, 2 days, 3 days, and 7 days after mTBI. Gray shaded areas depict comparison Athlete cohort mTBI and NC ≤6h timepoint comparisons for testing the null hypothesis with other Athlete cohort first week timepoints (2 day, 3 day, and 7 day). **Training/Discovery** = uses logistic regression analysis. **mTBI** = mild traumatic brain injury. **NC** = non-concussed teammate controls. **CI** = confidence interval. **SVM** = support vector machine. **LASSO** = least absolute shrinkage and selection operator. **Internal Validation** = uses logistic regression with 10-fold cross validation analysis. **Replication** = uses logistic regression analysis. **ROC AUC** = receiver operating characteristic area under the curve. **sens/spec** = sensitivity/specificity. **MS/MS 6** = final metabolite panel confirmed via tandem mass spectrometry (MS/MS). *No statistically significant difference in ROC AUC values when compared to shaded values in same row, per Hanley-McNeil test. Statistical significance considered if p <0.05.
(DOCX)

S5 Table Classification comparisons of preliminary and final biomarker panels between athlete and external cohorts, without and with batch correction adjustment. Gray shaded areas depict comparison values, within the same row, for testing the null hypothesis via Hanley-McNeil test between the Athlete and External cohort ROC AUC results. **ROC AUC** = receiver operating characteristic area under the curve. **SVM** = support vector machine. **LASSO** = least absolute shrinkage and selection operator. **sens/spec** = sensitivity/specificity. **Training/Discovery** = uses logistic regression analysis. **Internal Validation** = uses logistic regression with 10-fold cross validation analysis. **Replication** = uses logistic regression analysis. **z** = Hanley-McNeil statistic. **p** = 2-tailed level of significance. **MS/MS** = Resulting 6 metabolites confirmed via tandem mass spectrometry (MS/MS). Statistical significance considered if p <0.05.
(DOCX)

## Abbreviations

AA: arachidonic acid
AUC: area under the curve
CI: confidence interval
CNS: central nervous system
CSF: cerebrospinal fluid
DHA: docosahexaenoic acid
ER: emergency room
LC-MS: liquid chromatography-mass spectrometry
LASSO: least absolute shrinkage and selection operator
LysoPC: lysophosphatidylcholine
MS/MS: tandem mass spectrometry
mTBI: mild traumatic brain injury
NC: non-concussed control
PE: phosphatidylethanolamine
PLA_2_: phospholipase A_2_
PLS-DA: partial least squares-discriminant analysis
POS: point of service
PUFA: polyunsaturated fatty acid
ROC: receiver operating characteristic
SVM: support vector machine
TBI: traumatic brain injury
TUDCA: tauroursodexoxycholic acid
