# Supplementary figures and images for: Plasma metabolomic biomarkers accurately classify acute mild traumatic brain injury from controls
Source: PLoS One. 2018 Apr 20;13(4):e0195318. doi: 10.1371/journal.pone.0195318 (PMC5909890; doi:10.1371/journal.pone.0195318)

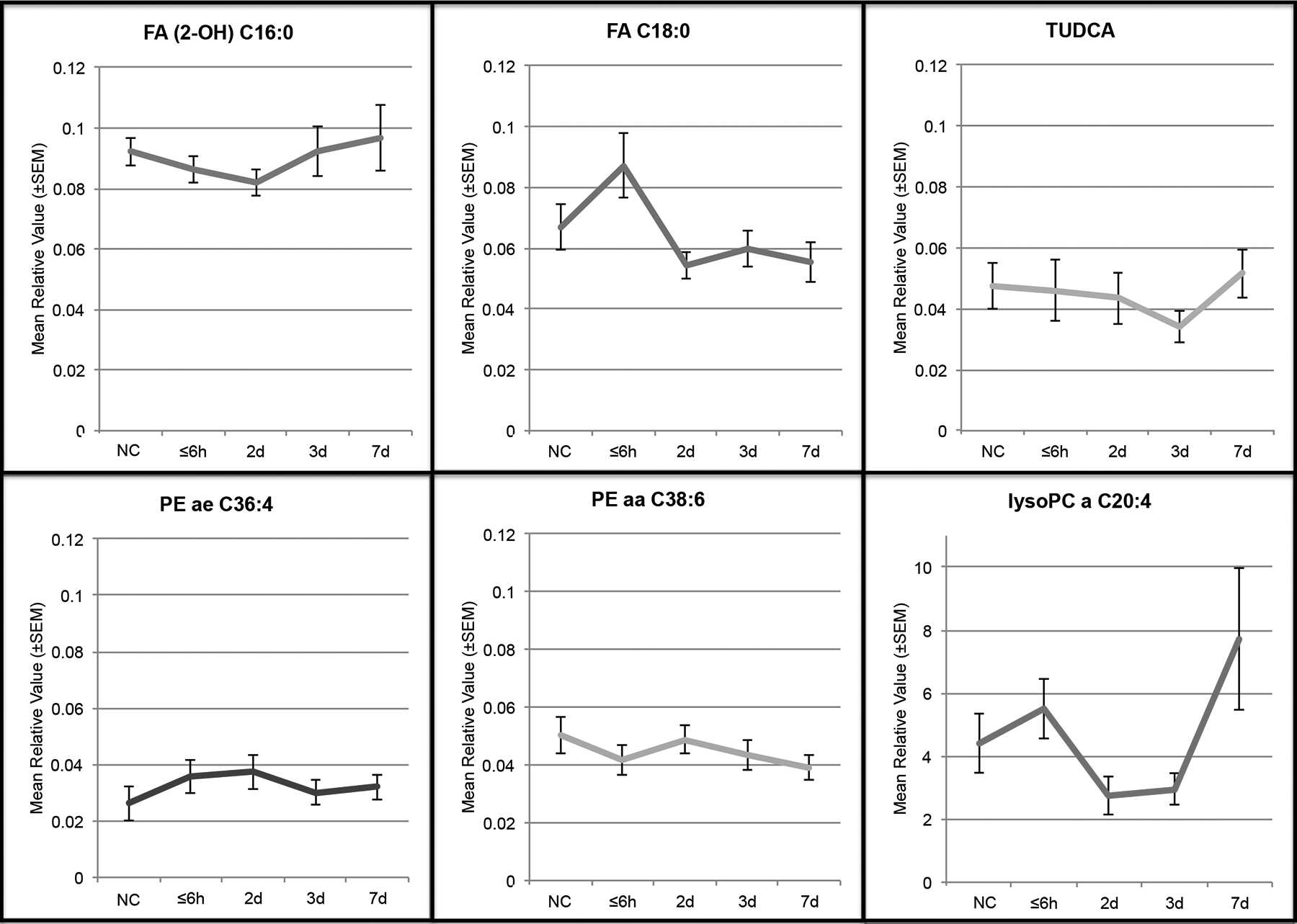

Supplement: S1 Fig — Individual mean metabolite relative values (RVs) ± SEM for Athlete NC and mTBI timepoints (≤6h, 2d, 3d, 7d) are plotted for each of the MS/MS confirmed metabolites. No statistically significant changes from NC values are noted for each metabolite, despite some fluctuations. NC = non-concussed controls. SEM = standard error of the mean. MS/MS = tandem mass spectrometry. (TIF) [file pone.0195318.s001.tif]

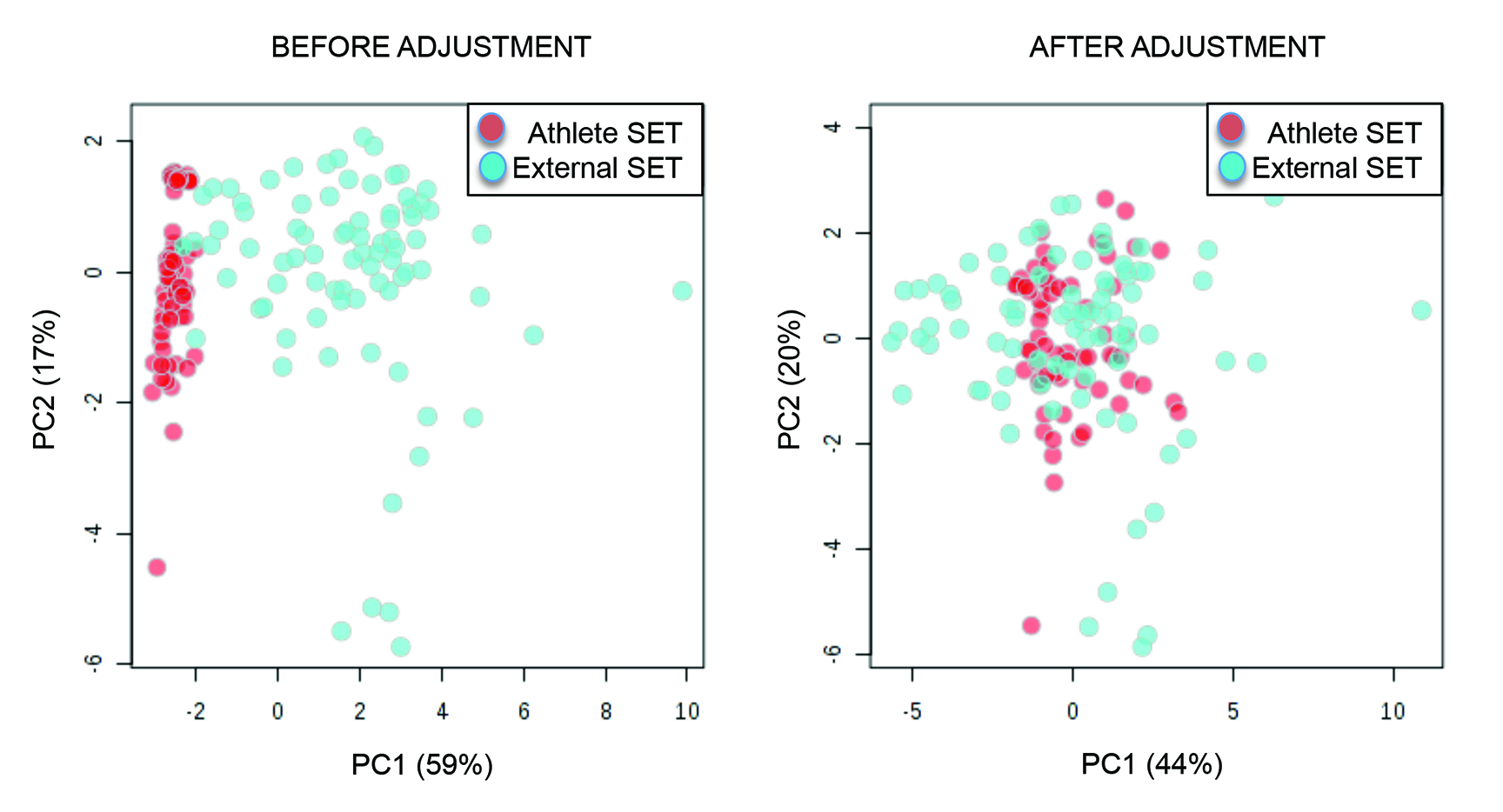

Supplement: S2 Fig — Note the dense clustering of Athlete cohort data in PC1 prior to Batch Correction (left) compared to after (right). Both Athlete and External cohorts appear more evenly distributed based on PC2, before and after adjustment. Although the Athlete and External cohort data overlap is improved with the Adjustment, the datasets continue to show differences. (TIF) [file pone.0195318.s002.tif]

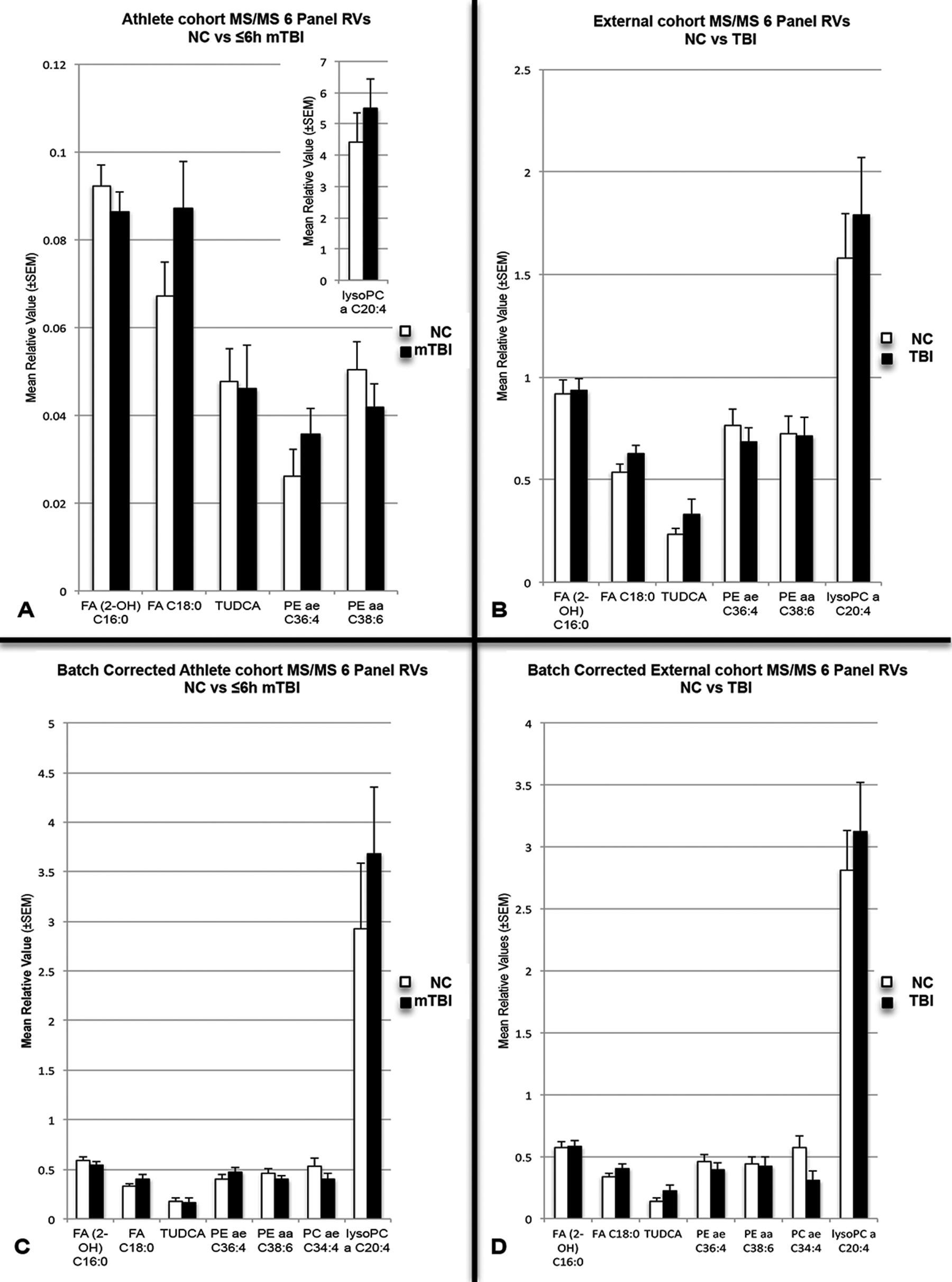

Supplement: S3 Fig — Mean relative values (RVs) ± SEM for NC and mTBI groups in the Athlete cohort at the ≤6h timepoint (A & C) and for the External cohort TBI and NC groups (B & D) are presented for each of the MS/MS-confirmed 6 plasma biomarkers. Note the relative improvement in quantitative comparability of metabolite RVs in both cohorts following Batch Correction (A vs. B; C vs. D). The individual analyte relative value (RV) differences did not reach statistical significance between the NC and mTBI (or TBI) groups, before or following batch correction. NC = non-concussed controls. TBI = traumatic brain injury. mTBI = mild TBI. SEM = standard error of the mean. MS/MS = tandem mass spectrometry. (TIF) [file pone.0195318.s003.tif]
