# Supplementary material for: Plasma metabolomic biomarkers accurately classify acute mild traumatic brain injury from controls
Source: PLoS One. 2018 Apr 20;13(4):e0195318. doi: 10.1371/journal.pone.0195318 (PMC5909890; doi:10.1371/journal.pone.0195318)
Supplement: S1 File — Athlete cohort discovery/internal validation specimen set. Note the complete QC pool overlay, and apparent consistency across all QCs. (PDF) [file pone.0195318.s004.pdf]

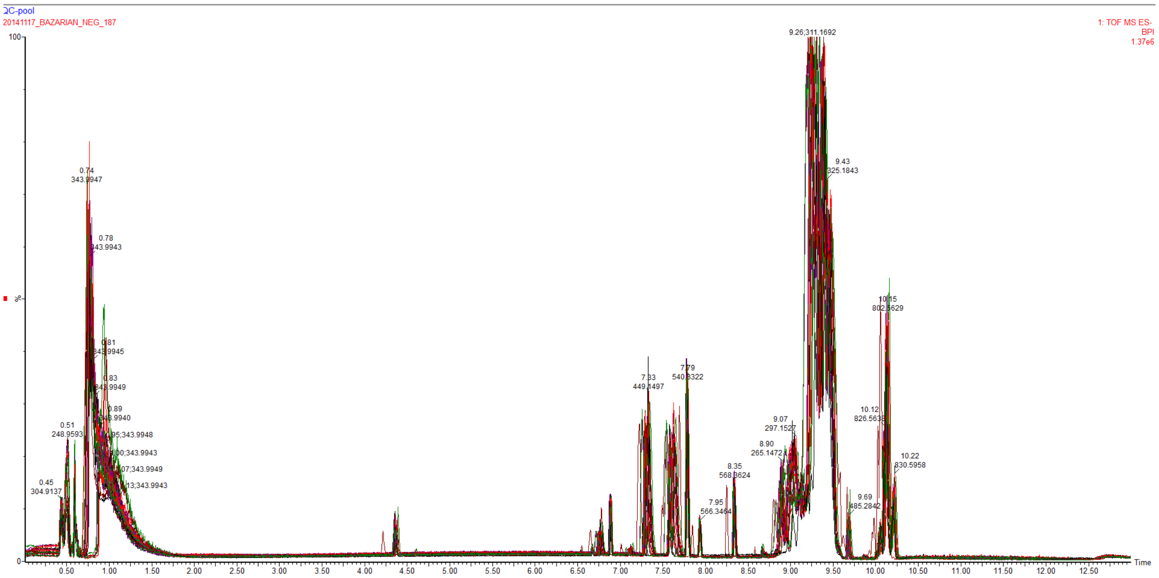

S1 File. Quality Control (QC) Total ion chromatogram – Negative mode. Athlete cohort discovery/internal validation specimen set. Note the complete QC pool overlay, and apparent consistency across all QCs.
