# Supplementary material for: Plasma metabolomic biomarkers accurately classify acute mild traumatic brain injury from controls
Source: PLoS One. 2018 Apr 20;13(4):e0195318. doi: 10.1371/journal.pone.0195318 (PMC5909890; doi:10.1371/journal.pone.0195318)

Validated as FA 2-OH C16:0; 2- Hydroxyhexadecanoic acid
m/z 271.2266

271.2273
225.2216
223.2057

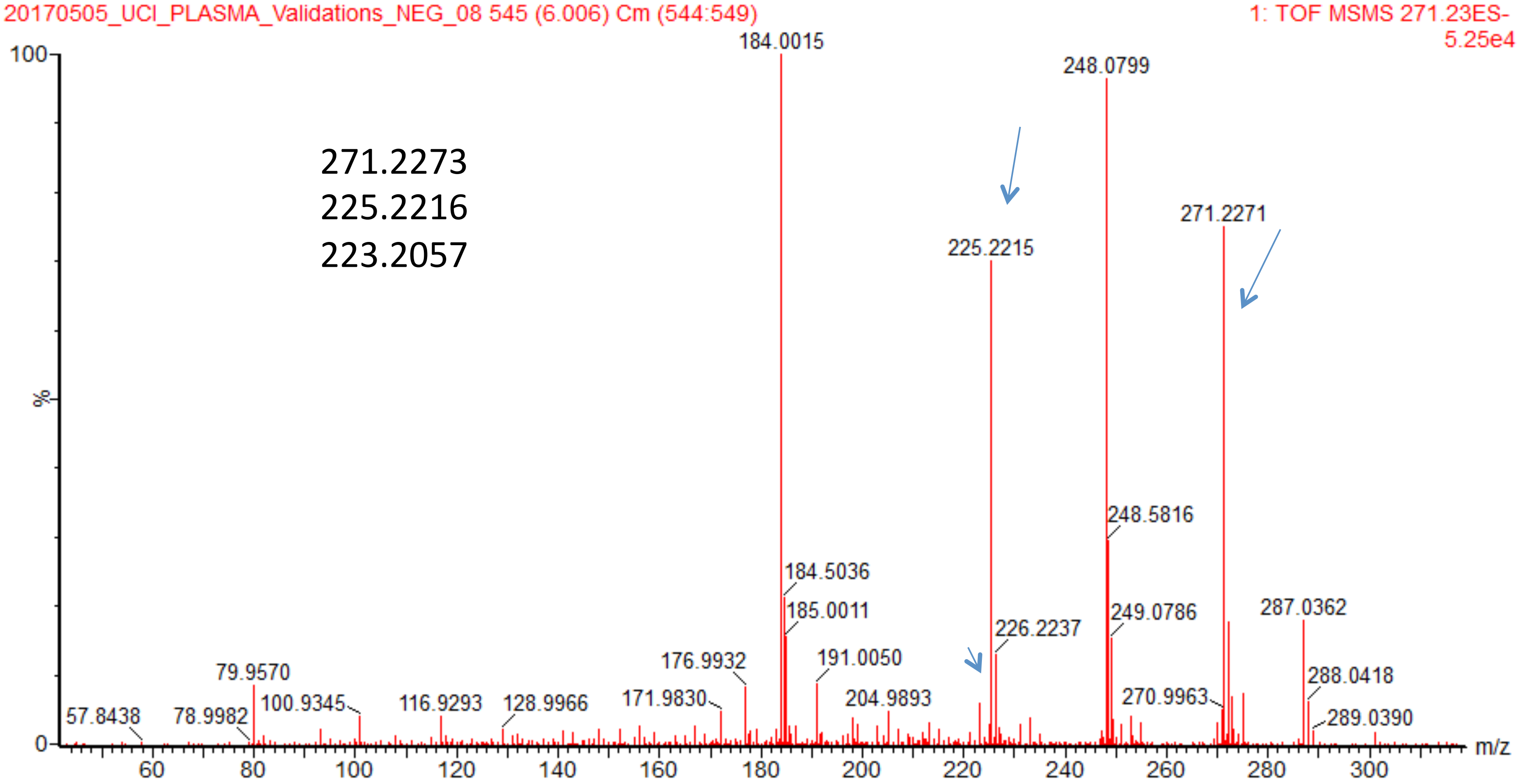

Validated as FA C18:0; Stearic acid
m/z 283.2629

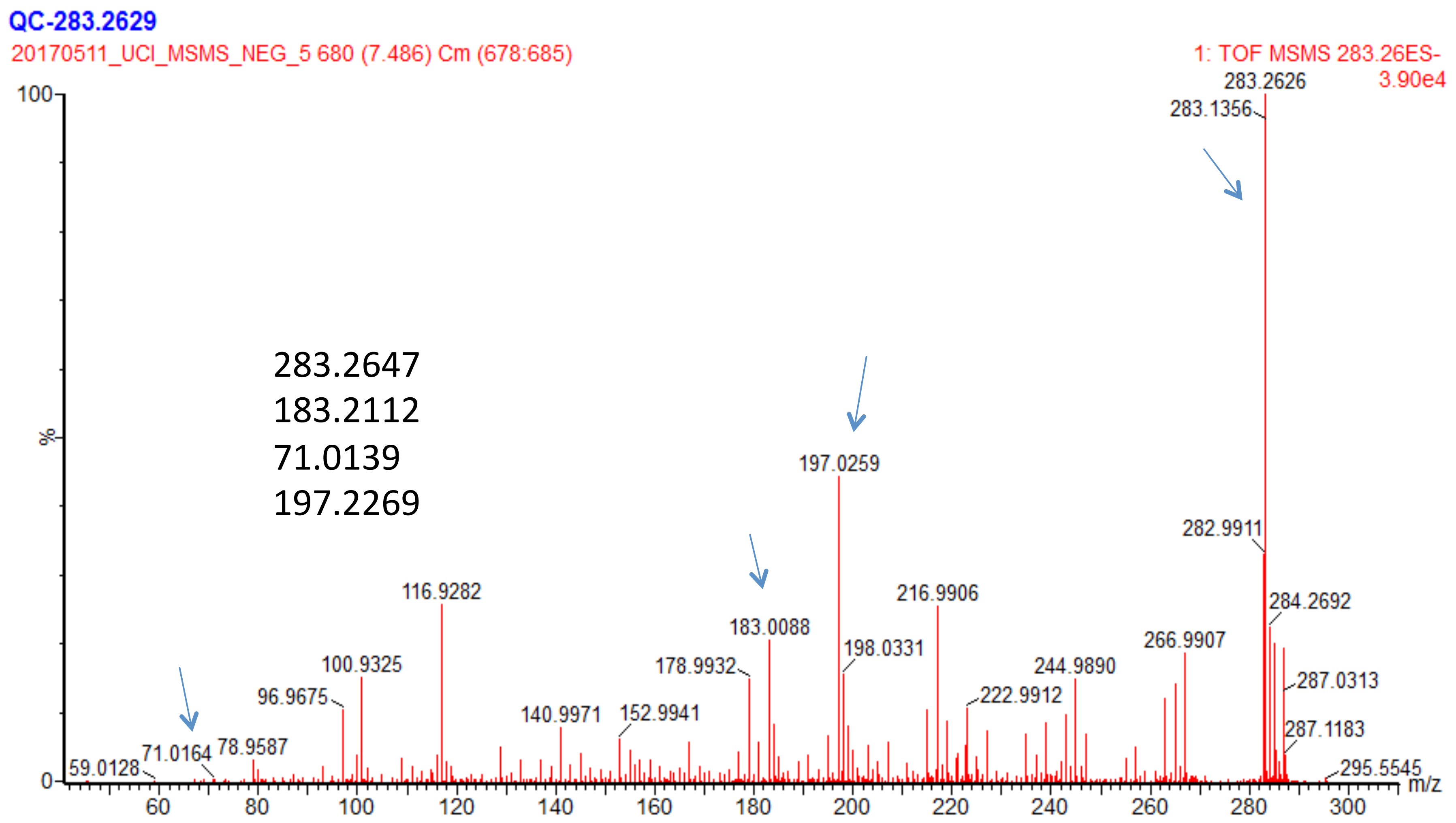

Validated as TUDCA; Tauroursodeoxycholic acid
m/z 498.2936

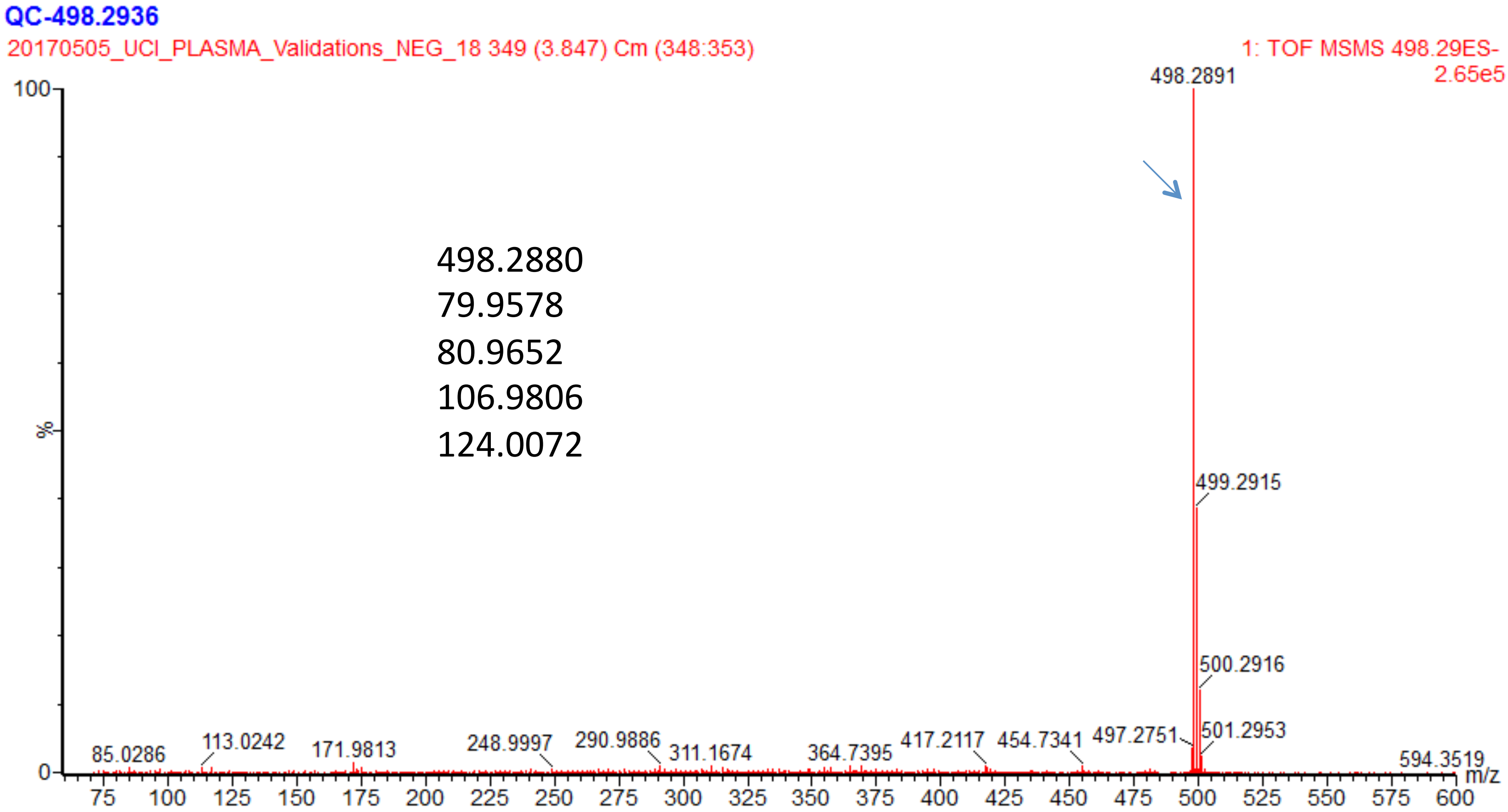

498.2880
79.9578
80.9652
106.9806
124.0072

Validated as PE ae C36:4; PE(P-16:0/20:4)
m/z 722.513

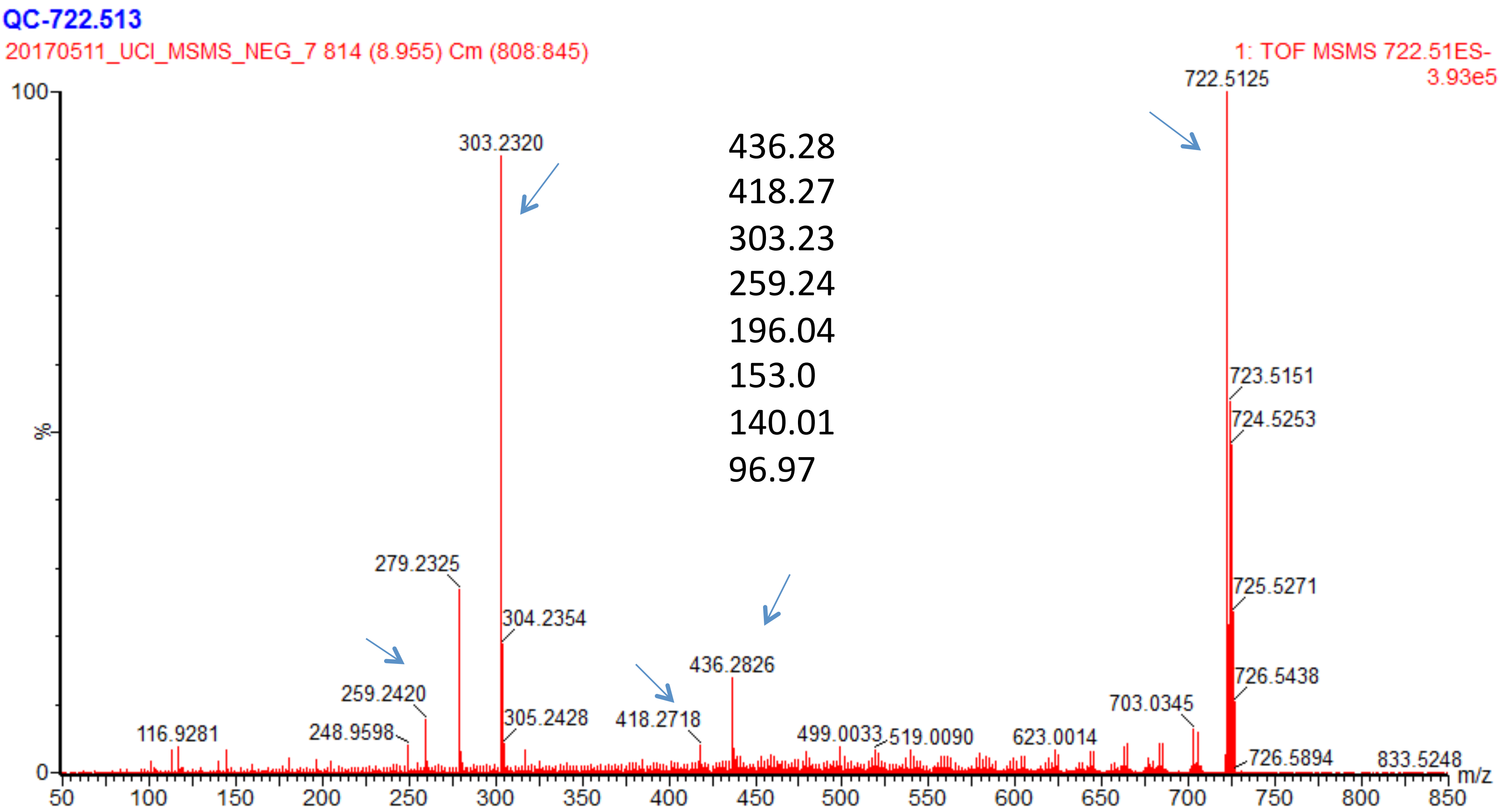

436.28
418.27
303.23
259.24
196.04
153.0
140.01
96.97

Validated as PE aa C38:6; PE(16:0/22:6)
m/z 762.5081

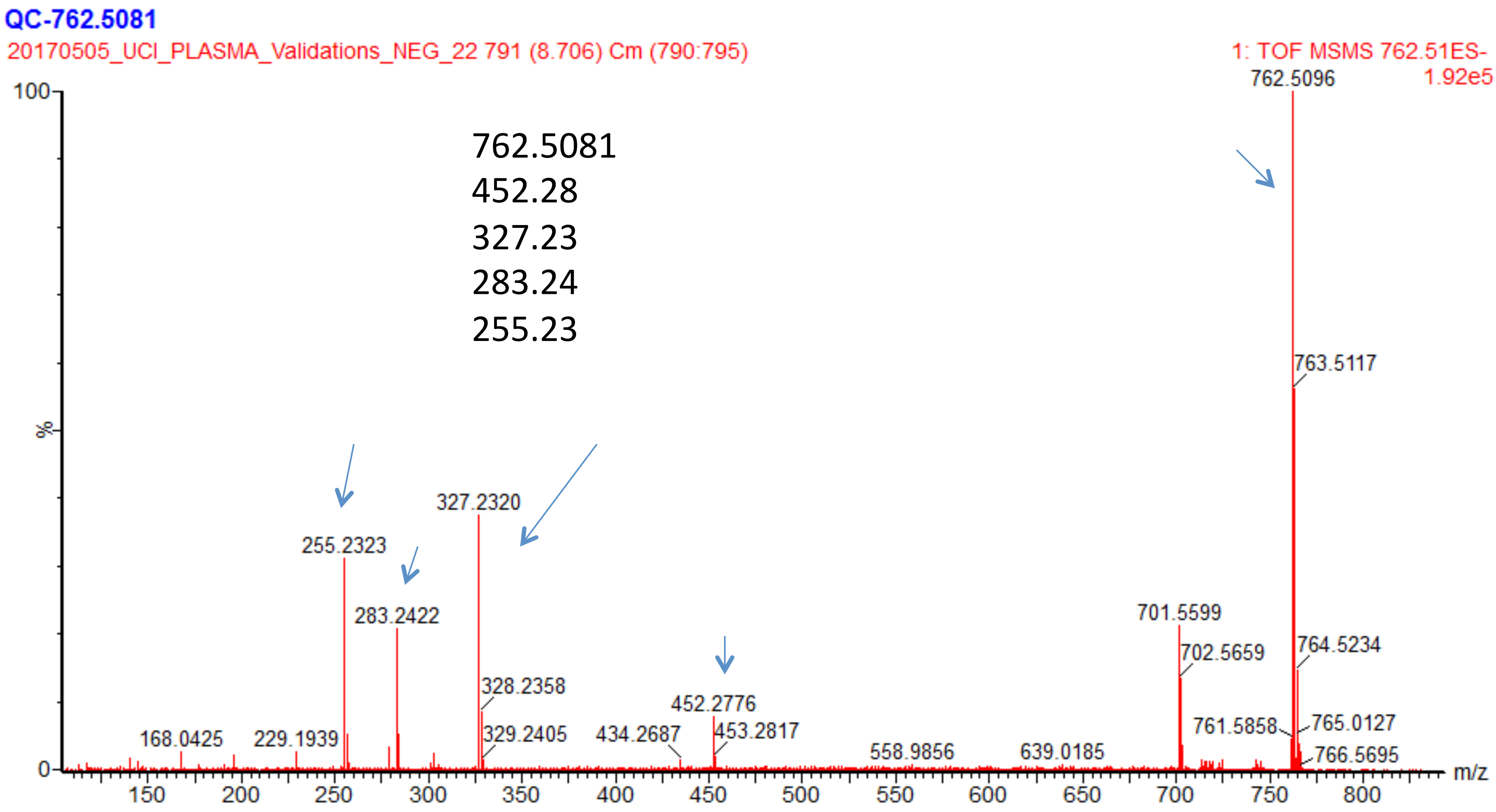

Validated as LysoPC a C20:4; PC(20:4/0:0)
m/z 544.3411

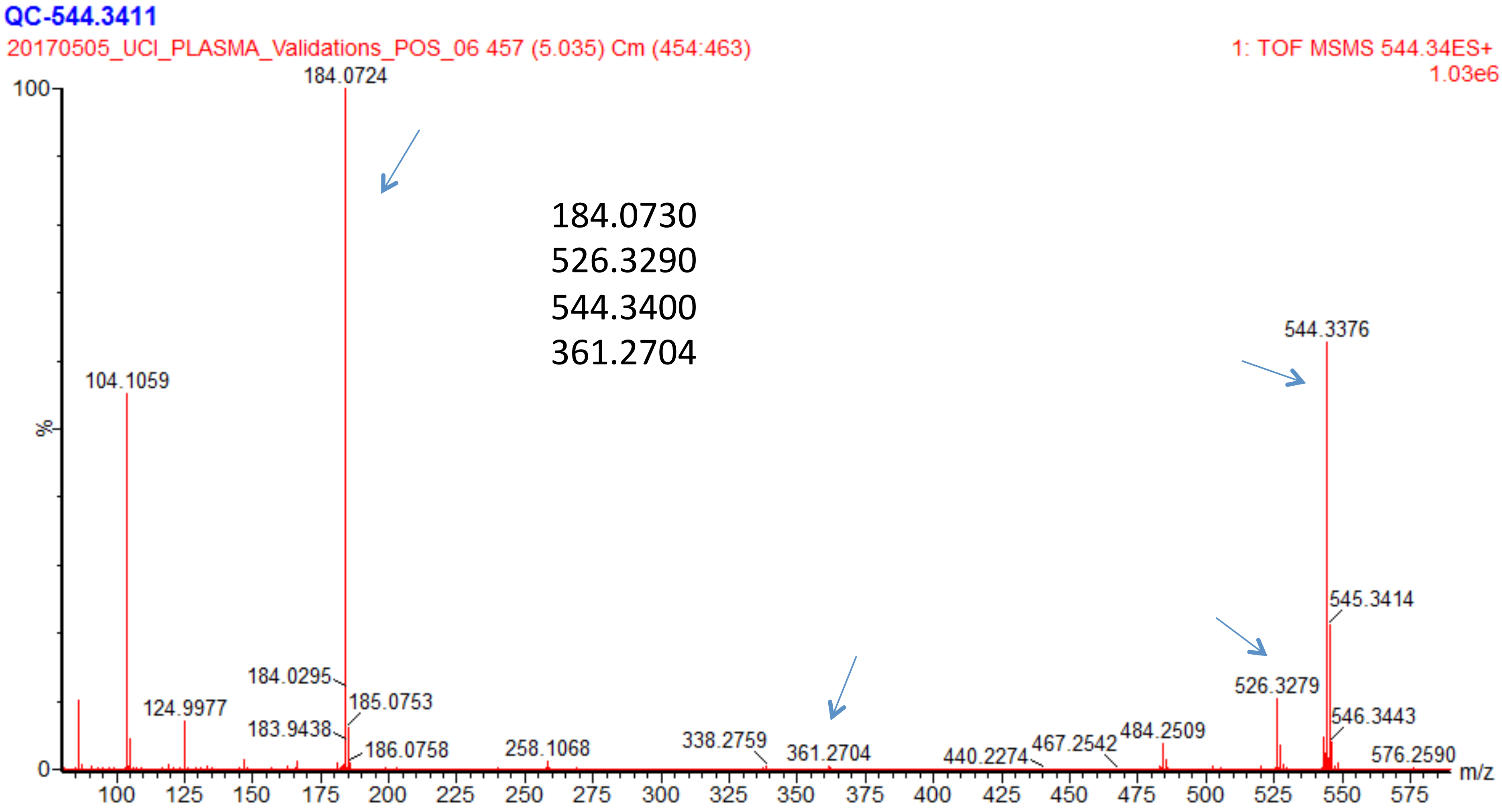

Supplement: S3 File — These six fragmentation spectra obtained from discovery specimens were matched with those known standards within the Human Metabolome or Lipid Maps Databases, using standard methods [38]. The six included spectra, therefore, confirmed our 6-metabolite panel that was discovered and internally validated within the Athlete cohort and replicated in the External cohort. (PDF) [file pone.0195318.s006.pdf]
