# Supplementary material for: Plasma metabolomic biomarkers accurately classify acute mild traumatic brain injury from controls
Source: PLoS One. 2018 Apr 20;13(4):e0195318. doi: 10.1371/journal.pone.0195318 (PMC5909890; doi:10.1371/journal.pone.0195318)
Supplement: S1 Table — mTBI = mild traumatic brain injury. NC = non-concussed teammate control. (DOCX) [file pone.0195318.s007.docx]

| **S1 Table. Athlete Cohort - Prior History of Traumatic Brain Injury** | | |
| --- | --- | --- |
| **Athlete Cohort** | **History of prior mTBI** | |
| **Groups (*n*)** | mTBI (38) | NC (24) |
| **With no history of previous concussion** | 29 (76%) | 14 (58%) |
| **With history of previous concussion** | 9 (24%) | 10 (42%) |
| **Yes, 1 prior concussion** | 4 | 7 |
| **Yes, >1 prior concussion** | 5 | 3 |
| mTBI = mild traumatic brain injury. NC = non-concussed teammate control. | | |
