# Supplementary material for: Plasma metabolomic biomarkers accurately classify acute mild traumatic brain injury from controls
Source: PLoS One. 2018 Apr 20;13(4):e0195318. doi: 10.1371/journal.pone.0195318 (PMC5909890; doi:10.1371/journal.pone.0195318)
Supplement: S2 Table — Shaded areas indicate models with best results when comparing ROC AUC values using various feature selection methods in the Athlete cohort. LR = logistic regression. CI = confidence interval. ROC = receiver operating characteristic. AUC = area under the curve. sens/spec = sensitivity/specificity. SVM = support vector machine. PLS-DA = partial least squares-discriminant analysis. LASSO = least absolute shrinkage and selection operator. Targeted 1 = selected based on highest-ranking metabolites AUC values in the Tester for analytes included in Biocrates AbsoluteIDQ® p180 Kit. Targeted 2 = selected based on the highest-ranking lipid AUC values in the Tester for analytes included in Biocrates AbsoluteIDQ® p180 Kit. (DOCX) [file pone.0195318.s008.docx]

| **S2 Table. Athlete Cohort Analysis Using Six Feature Selection-Derived Models** | | | |
| --- | --- | --- | --- |
| **Feature Selection Method** | **Number of Analytes (Features) In Panel** | **Athlete Cohort Training/Discovery LR**  **ROC AUC**  **(95% CI)**  (sens/spec) | **Athlete Cohort**  **Internal Validation**  **LR+10-fold cross validation**  **ROC AUC**  **(95% CI)**  (sens/spec) |
| **Linear SVM** | **10** | **0.976**  **(0.965-0.988)**  (0.778/1.00) | **0.864**  **(0.750-0.978)**  (0.815/0.917) |
| **PLS-DA** | **10** | **0.928**  **(0.907-0.949)**  (0.733/0.981) | **0.766**  **(0.627-0.906)**  (0.704/0.833) |
| **Random Forests** | **10** | **0.918**  **(0.895-0.942)**  (0.630/1.00) | **0.752**  **(0.609-0.895)**  (0.667/0.972) |
| **LASSO** | **10** | **0.974**  **(0.963-0.985)**  (0.663/1.00) | **0.865**  **(0.771-0.960)**  (0.704/0.944) |
| **Targeted 1** | **10** | **0.807**  **(0.771-0.843)**  (0.576/0.898) | **0.575**  **(0.420-0.730)**  (0.519/0.694) |
| **Targeted 2** | **14** | **0.944**  **(0.926-0.962)**  (0.942/0.824) | **0.636**  **(0.487-0.785)**  (0.704/0.694) |
| Shaded areas indicate models with best results when comparing ROC AUC values using various feature selection methods in the Athlete cohort. **LR** = logistic regression. **CI** = confidence interval. **ROC** = receiver operating characteristic. **AUC** = area under the curve. **sens/spec** = sensitivity/specificity. **SVM** = support vector machine. **PLS-DA** = partial least squares-discriminant analysis. **LASSO** = least absolute shrinkage and selection operator. **Targeted 1** = selected based on highest-ranking metabolites AUC values in the *Tester* for analytes included in Biocrates *AbsoluteIDQ®* p180 Kit. **Targeted 2** = selected based on the highest-ranking lipid AUC values in the *Tester* for analytes included in Biocrates *AbsoluteIDQ®* p180 Kit. | | | |
