# Supplementary material for: Plasma metabolomic biomarkers accurately classify acute mild traumatic brain injury from controls
Source: PLoS One. 2018 Apr 20;13(4):e0195318. doi: 10.1371/journal.pone.0195318 (PMC5909890; doi:10.1371/journal.pone.0195318)
Supplement: S3 Table — Gray shaded area depicts comparison Season Athlete ≤6h internal validation values for testing the null hypothesis on External cohort Replication ROC AUC results from each of the mTBI and >mTBI groups. CI = confidence interval. MS/MS 6 = Final six metabolite panel confirmed via tandem mass spectrometry (MS/MS). ROC = receiver operating characteristic. AUC = area under the curve. sens/spec = sensitivity/specificity. Training/Discovery = uses logistic regression analysis. Internal Validation = uses logistic regression with 10-fold cross validation analysis. Replication = uses logistic regression analysis. NC = non-concussed controls. mTBI = mild traumatic brain injury. >mTBI = TBI noted to be worse than mTBI, including mTBI with abnormal MRI, moderate TBI, or severe TBI. *No statistically significant difference when compared to shaded value in same row, via Hanley-McNeil test. Statistical significance considered if p <0.05. (DOCX) [file pone.0195318.s009.docx]

| **S3 Table. MS/MS 6 Panel Classification Accuracy for the TBI Severity Groups** | | | | | |
| --- | --- | --- | --- | --- | --- |
| **Feature Selection Method** | **Athlete Cohort**  **Season ≤6h mTBI versus Season NC Training/Discovery**  **ROC AUC**  **(95% CI)**  (sens/spec) | **Athlete Cohort Season ≤6h mTBI versus Season NC Internal Validation**  **ROC AUC**  **(95% CI)**  (sens/spec) | **External Cohort mTBI versus NC**  **Replication**  **ROC AUC**  **(95% CI)**  (sens/spec) | **External Cohort >mTBI versus NC**  **Replication**  **ROC AUC**  **(95% CI)**  (sens/spec) | |
| **MS/MS 6** | **0.847**  **(0.815-0.879)** (0.770/0.784) | **0.791**  **(0.677-0.905)** (0.741/0.778) | **0.751***  **(0.707-0.795)**  (0.628/0.740) | | **0.768***  **(0.724-0.811)**  (0.869/0.597) |
| Gray shaded area depicts comparison Season Athlete ≤6h internal validation values for testing the null hypothesis on External cohort Replication ROC AUC results from each of the mTBI and >mTBI groups. **CI** = confidence interval. **MS/MS 6** = Final six metabolite panel confirmed via tandem mass spectrometry (MS/MS). **ROC** = receiver operating characteristic. **AUC** = area under the curve. **sens/spec** = sensitivity/specificity. **Training/Discovery** = uses logistic regression analysis. **Internal Validation** = uses logistic regression with 10-fold cross validation analysis. **Replication** = uses logistic regression analysis. **NC** = non-concussed controls. **mTBI** = mild traumatic brain injury. **>mTBI** = TBI noted to be worse than mTBI, including mTBI with abnormal MRI, moderate TBI, or severe TBI. *No statistically significant difference when compared to shaded value in same row, via Hanley-McNeil test. Statistical significance considered if p <0.05. | | | | | |
