# Supplementary material for: Plasma metabolomic biomarkers accurately classify acute mild traumatic brain injury from controls
Source: PLoS One. 2018 Apr 20;13(4):e0195318. doi: 10.1371/journal.pone.0195318 (PMC5909890; doi:10.1371/journal.pone.0195318)
Supplement: S4 Table — Gray shaded areas depict comparison Athlete cohort mTBI and NC ≤6h timepoint comparisons for testing the null hypothesis with other Athlete cohort first week timepoints (2 day, 3 day, and 7 day). Training/Discovery = uses logistic regression analysis. mTBI = mild traumatic brain injury. NC = non-concussed teammate controls. CI = confidence interval. SVM = support vector machine. LASSO = least absolute shrinkage and selection operator. Internal Validation = uses logistic regression with 10-fold cross validation analysis. Replication = uses logistic regression analysis. ROC AUC = receiver operating characteristic area under the curve. sens/spec = sensitivity/specificity. MS/MS 6 = final metabolite panel confirmed via tandem mass spectrometry (MS/MS). *No statistically significant difference in ROC AUC values when compared to shaded values in same row, per Hanley-McNeil test. Statistical significance considered if p <0.05. (DOCX) [file pone.0195318.s010.docx]

| **S4 Table. Biomarker Panel Classification in the Athlete Cohort at ≤6h, 2 days, 3 days, and 7 days after mTBI** | | | | | |
| --- | --- | --- | --- | --- | --- |
| **Biomarker Panel** | **Season ≤6h mTBI**  **Training/Discovery**  **ROC AUC**  **(95% CI)**  (sens/spec) | **Season ≤6h mTBI**  **Internal Validation**  **ROC AUC**  **(95% CI)**  (sens/spec) | **Season 2 day mTBI Replication**  **ROC AUC**  **(95% CI)**  (sens/spec) | **Season 3 day mTBI Replication**  **ROC AUC**  **(95% CI)**  (sens/spec) | **Season 7 day mTBI Replication**  **ROC AUC**  **(95% CI)**  (sens/spec) |
| **Linear SVM 6** | **0.913**  **(0.888-0.938)** (0.835/0.907) | **0.851**  **(0.745-0.957)** (0.815/0.861) | **0.910***  **(0.883-0.936)**  (0.840/0.938) | **0.853***  **(0.820-0.886)**  (0.760/0.877) | **0.953***  **(0.939-0.968)**  (0.811/0.957) |
| **LASSO 8** | **0.948**  **(0.930-0.965)**  (0.852/0.914) | **0.848**  **(0.746-0.949)**  (0.741/0.861) | **0.960***  **(0.945-0.974)**  (0.866/0.944) | **0.905***  **(0.880-0.931)**  (0.750/0.957) | **0.942***  **(0.923-0.961)**  (0.826/0.975) |
| **MS/MS 6** | **0.847**  **(0.815-0.879)** (0.770/0.784) | **0.791**  **(0.677-0.905)** (0.741/0.778) | **0.815***  **(0.781-0.848)**  (0.725/0.778) | **0.707***  **(0.667-0.748)**  (0.510/0.725) | **0.716***  **(0.677-0.755)**  (0.712/0.586) |
| Gray shaded areas depict comparison Athlete cohort mTBI and NC ≤6h timepoint comparisons for testing the null hypothesis with other Athlete cohort first week timepoints (2 day, 3 day, and 7 day). **Training/Discovery** = uses logistic regression analysis. **mTBI** = mild traumatic brain injury. **NC** = non-concussed teammate controls. **CI** = confidence interval. **SVM** = support vector machine. **LASSO**  = least absolute shrinkage and selection operator. **Internal Validation** = uses logistic regression with 10-fold cross validation analysis. **Replication** = uses logistic regression analysis. **ROC AUC** = receiver operating characteristic area under the curve. **sens/spec** = sensitivity/specificity. **MS/MS 6** = final metabolite panel confirmed via tandem mass spectrometry (MS/MS). *No statistically significant difference in ROC AUC values when compared to shaded values in same row, per Hanley-McNeil test. Statistical significance considered if p <0.05. | | | | | |
