# Supplementary material for: Plasma metabolomic biomarkers accurately classify acute mild traumatic brain injury from controls
Source: PLoS One. 2018 Apr 20;13(4):e0195318. doi: 10.1371/journal.pone.0195318 (PMC5909890; doi:10.1371/journal.pone.0195318)
Supplement: S5 Table — Gray shaded areas depict comparison values, within the same row, for testing the null hypothesis via Hanley-McNeil test between the Athlete and External cohort ROC AUC results. ROC AUC = receiver operating characteristic area under the curve. SVM = support vector machine. LASSO = least absolute shrinkage and selection operator. sens/spec = sensitivity/specificity. Training/Discovery = uses logistic regression analysis. Internal Validation = uses logistic regression with 10-fold cross validation analysis. Replication = uses logistic regression analysis. z = Hanley-McNeil statistic. p = 2-tailed level of significance. MS/MS = Resulting 6 metabolites confirmed via tandem mass spectrometry (MS/MS). Statistical significance considered if p <0.05. (DOCX) [file pone.0195318.s011.docx]

| **S5 Table. Classification Comparisons of Preliminary and Final Biomarker Panels Between Athlete and External Cohorts, without and with Batch Correction Adjustment** | | | | | |
| --- | --- | --- | --- | --- | --- |
| **Feature Selection Method Defining Panel** | **No. of analytes in panel** | **Athlete Cohort Training/Discovery**  **ROC AUC**  **(95% CI)**  (sens/spec) | **Athlete Cohort**  **Internal Validation**  **ROC AUC**  **(95% CI)**  (sens/spec) | **External Cohort**  **Replication**  **ROC AUC**  **(95% CI)**  (sens/spec) | **Hanley-McNeil Test Results**  **(z, p)** |
| **Linear SVM** | 6 | **0.913**  **(0.888-0.938)** (0.835/0.907) | **0.851**  **(0.745-0.957)** (0.815/0.861) | **0.830**  **(0.798-0.861)**  (0.817/0.715) | **0.293, 0.770** |
|  | 6 (with batch correction) | **0.911**  **(0.886-0.936)**  (0.774/0.944) | **0.859**  **(0.755-0.963)**  (0.778/0.944) | **0.758**  **(0.719-0.796)**  (0.724/0.751) | **1.325, 0.185** |
| **LASSO** | 8 | **0.948**  **(0.930-0.965)**  (0.852/0.914) | **0.848**  **(0.746-0.949)**  (0.741/0.861) | **0.811**  **(0.781-0.841)**  (0.778/0.686) | **0.502, 0.616** |
|  | 8 (with batch correction) | **0.938**  **(0.919-0.957)**  (0.704/0.991) | **0.828**  **(0.718-0.938)**  (0.741/0.889) | **0.763**  **(0.729-0.796)**  (0.642/0.736) | **0.822, 0.411** |
| **MS/MS** | 6 | **0.847**  **(0.815-0.879)** (0.770/0.784) | **0.791**  **(0.677-0.905)** (0.741/0.778) | **0.738**  **(0.703-0.773)** (0.695/0.644) | **0.633, 0.527** |
|  | 6 (with batch correction) | **0.845**  **(0.811-0.879)**  (0.823/0.787) | **0.775**  **(0.656-0.893)**  (0.741/0.750) | **0.768**  **(0.735-0.801)**  (0.659/0.665) | **0.839, 0.933** |
| Gray shaded areas depict comparison values, within the same row, for testing the null hypothesis via Hanley-McNeil test between the Athlete and External cohort ROC AUC results. **ROC AUC** = receiver operating characteristic area under the curve. **SVM** = support vector machine. **LASSO**  = least absolute shrinkage and selection operator. **sens/spec** = sensitivity/specificity. **Training/Discovery** = uses logistic regression analysis. **Internal Validation** = uses logistic regression with 10-fold cross validation analysis. **Replication** = uses logistic regression analysis. **z** = Hanley-McNeil statistic. **p** = 2-tailed level of significance. **MS/MS** = Resulting 6 metabolites confirmed via tandem mass spectrometry (MS/MS). Statistical significance considered if p <0.05. | | | | | |
